# Auxotrophic mutations of *Trichophyton rubrum* created by in vitro synthesized Cas9 ribonucleoprotein

**DOI:** 10.1186/s12896-020-0601-z

**Published:** 2020-01-20

**Authors:** Oliver Blechert, Huan Mei, Xiaohui Zang, Hailin Zheng, Guanzhao Liang, Weida Liu

**Affiliations:** 1 Department of Medical Mycology, Institute of Dermatology, Chinese Academy of Medical Science and Peking Union Medical College, Nanjing, Jiangsu 210042 People’s Republic of China; 2 Jiangsu Key Laboratory of Molecular Biology for Skin Diseases and STIs, Nanjing, Jiangsu 210042 People’s Republic of China; 3 Center for Global Health, School of Public Health, Nanjing Medical University, Nanjing, Jiangsu 211166 People’s Republic of China

**Keywords:** *Trichophyton rubrum*, Cas9 ribonucleoprotein complex, Gene knock-out, Uracil, Tryptophan

## Abstract

**Background:**

*Trichophyton rubrum* is an obligate human parasitic fungus and responsible for approximately 80–90% of dermatomycosis in human. Molecular genetic manipulations of this pathogen are challenging and available tools and protocols are only rudimentary. We adapt molecular genetics methods of well established fungal model organism, to knock out genes in *T. rubrum*. For the adaptation, crucial modifications are necessary. With the implementation of in vitro synthesized Cas9-sgRNA ribonucleoprotein complex, it is possible to adapt molecular genetic methods, to knock out genes in *T. rubrum*.

**Results:**

The gene knock-out method is based on integration of a selection marker into the target site, to interrupt the gene translation. The target gene gets preassigned by the homologous sequence of the in vitro synthesized Cas9-sgRNA ribonucleoprotein complex. To develop the method, we first isolated and characterized a *T. rubrum* strain with a high amount of microconidia. Next, we developed a transformation protocol, whereby the Cas9-sgRNA ribonucleoprotein gets delivered into the fungal protoplast by the PEG method.

We knocked out the URA3 gene and resulted, as predicted, uracil auxotrophic strains. These strains can be used for specific gene knock-outs by reintegrating the URA3 fragment and selection on uracil lacking cultivation media. Exemplary, we knocked out the TRP3 gene and got the predicted phenotype, tryptophan auxotrophic strains. The mutation had been verified by sequencing.

**Conclusions:**

We developed a method, based on in vitro synthesized Cas9-sgRNA ribonucleoprotein complex, for target specific gene knock-outs in *T. rubrum*. We knocked out the *Ura3* gene and resulted uracil auxotrophic strains. These strains were used for target specific gene knock-outs by reintegrating the *Ura3* fragment into the target gene site to interrupt the gene transcription. The developed method allows to adapt sophisticate gene manipulation methods of model fungal species to non-model species.

## Background

*Trichophyton rubrum* (Castellani) Sabouraud 1911 is a haploid ascomycetes fungus of the order *Onygenales*. As an obligate human pathogenic fungus, *T. rubrum* is in the focus of medicine and research for more than a century. Moreover, in the last decades the fungus got the most relevant dermatophyte and is responsible for 80–90% of the dermatomycosis cases. Contrary, only limited genetic informations are available and the development of genetic tools and protocols are far behind in comparison to other medical important fungi as *Candida albicans* and *Aspergillus* spp. [1].

In fungal model-organism auxotrophic selection-markers are a standard tool for selecting transformed colonies. Widely used is the orotidine-5′-monophosphate decarboxylase gene, in *Saccharomyces cerevisiae* with the standard name *URA3*. The *URA3* gene is part of the uracil pathway and knock down of the gene causes an auxotrophy, which can be compensated by supplying uracil (Ura) or uridine (Uri). A special advantage of using the *URA3* gene as selection-marker is the possibility of counter-selection by 5-fluoroorotic acid (FOA) [2]. *S. cerevisiae* and *C. albicans* ∆*ura3* strains can not grow in the absence of uracil, but can grow on FOA media, whereas the wild type can grow in the absence of uracil but not on FOA media.

Integration of a DNA fragment into the genome of a fungus takes place at a double string break (DSB) sites in a chromosome. These breaks can be caused randomly by UV radiation or restriction enzymes. In *Trichophyton mentagrophytes* the restriction enzyme mediated integration (REMI) had been used to tag the fungus with a GFP signal [3]. When a DNA fragment gets integrated into the genome, basically two different types of repair mechanisms are involved. The first is the homologous directed repair (HDR) mechanism of the fungus. For this, the integration fragment must be designed with sequences that are homologous to both flanking sites. The DSB is repaired by homologous recombination and the fragment will be integrated at the target site. In *S. cerevisiae* and *C. albicans* genome manipulation by homologous recombination is the most commonly applied method. Since in *Trichophyton* spp*.,* DNA fragments are dominantly integrated by the non-homologous end joining (NHEJ) repair mechanism, and homologous integration is inhibited. By knocking out the *KU80* gene, which is a key factor of NHEJ, the rate of homologous integration increased in *T. mentagraphitis* from lower than 3% to approximately 70% [4].

The Cas9 protein allows to induce target specific DSBs. The Cas9 protein is guided by a sgRNA fragment to the specific site. Commonly the sgRNA consist of a 17-20 bp long protospacer region and a 80 bp long tail. The tail has a specific loop structure which is required for the ribonucleoprotein complex formation. The protospacer region is responsible for docking to a specific site at the chromosome [5]. By changing the protospacer sequence, homologous to the genome target sequence, DSBs can be induced in nearly every gene. In the well studied fungal genus *Aspergillus*, the Cas9 gene had been expressed from an autonomous replication vector [6]. In fungal species, which are not so well studied, and especially when no autonomous replication vectors are available, the Cas9 can be delivered as in vitro synthesized ribonucleoprotein complex instead of expressing both components from vectors. The method had been applied to *Mucor circinelloides* [7] and *Fusarium oxysporum* [8] and the ribonucleoprotein complex had been delivered into the cell by the PEG method.

In this study, we developed for the first time a transformation system for *T. rubrum*, based on delivering the Cas9-sgRNA ribonucleoprotein complex together with the *URA3* gene DNA fragment into the protoplast by the PEG method. For this, we have isolated and characterized a suitable monoclonal wild-type strain and knocked out the *URA3* gene. One of the ∆ura3 strains had been used to create a target specific knock-out based on the uracil selection method.

## Results

### Fungal strains

The two fungal strains, STRB008 and STRB012 were derived from clinical strains. After two rounds of single colony selection, the strains were preserved in September 2017 at − 80 °C. We tested the storage in 20% glycerol solution and the storage of conidia suspension dropped on filter disks. With both methods the fungi were recovered after one year, even after repeated freeze and thaw cycles.

**Fig. 1:**
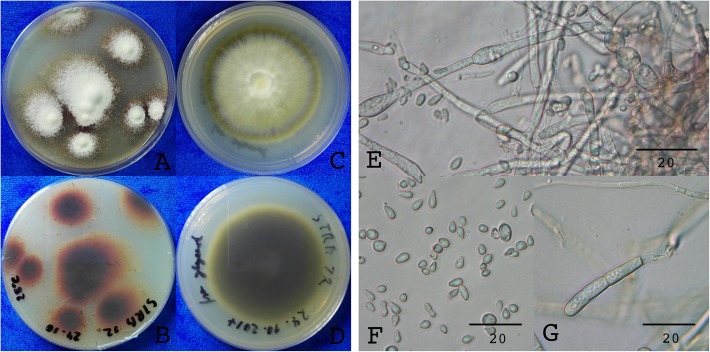
Phenotype and morphology of *T. rubrum* STRB012. **a**, **b**. Three weeks old culture grown on PDA agar. The red pigmentation is strongly developed. **c**, **d** Three weeks old culture grown on SC agar. The colony has a yellow to brownish pigmentation. **e**. Hyphae, microconidia and chlamydospores. **f** Microconidia. **g** Macroconidium

**Fig. 2:**
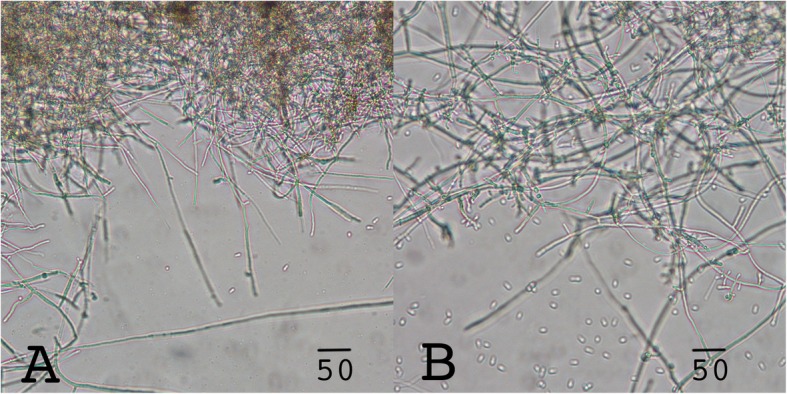
Induction of microconidia by CO_2_. **a** Three weeks old culture grown on Tr1 media at 28 °C. G. same as **b** but supplied with 5% CO_2_ to induce conidiogenesis

The fungi were identified by phenotype, microscopic analysis and comparison of the internal transcribed spacer (ITS) sequence. On PDA plates STRB012 developed the typical red pigments (Fig. 1a, b), whereas on SC medium the fungus had a yellow to brownish color (Fig. 1c, d). Hyphae of STRB012 were septate, 3.5–4 μm in diameter, infrequent curved and typically branched in right angle. Chlamydospores were in short chains, rounded to oval and 6–10 × 7–8 μm (Fig. 1e). The microconidia were rounded to pear-shaped, flattened at the narrowed side and 4–6 × 2–3 μm (Fig. 1f). The macroconidia were septate, predominately in three cells, with a size of 28–45 × 4–5 μm (Fig. 1g). Several microconidia developed two weeks after incubation at 28 °C on Tr1 medium. The development of microconidia could be stimulated by increasing the CO_2_ level to 5% (Fig. 2a, b).

The ITS1 and 2 sequences were identical to the *T. rubrum* CBS 392.58, except of one nucleotide in strain STRB012. The ITS 1 sequence of CBS 392.58 (NCBI NR_131330.1) has a cytosine at position 213 instead of thymine (see Additional file 1).

### Knock-out of the uracil pathway

For each of the knock-out experiments, two petri dishes, with Tr1 medium, were inoculated with STRB012. The cultures had been incubated at 28 °C with 5% CO_2_ for three weeks. Conidia were harvested by overlaying the plates with liquid SC medium and gently scratching the surface with a spreader. The conidia dispersions were filtrated through Miracloth to remove hyphal fragments. The purified conidia dispersion were filled up to 20 ml and shaken at 28 °C for 20 h with 120 rpm to induce germination. Afterwards, the liquid media were removed by centrifuging with 13,523 g for 10 min and washed twice with KCl buffer. The conidia were resuspended in 5 ml lysis buffer and incubated for 200 min at 28 °C with shaking. The cell wall digested conidia were centrifuged for 10 min with 9391 g and washed twice with KCl buffer. A total amount of 10 million protoplasts were resuspended in 250 μl sucrose buffer by carefully flicking the reaction cup.

Each 100 μl of the protoplast dispersion were added to 35 μl transformation solution, gently mixed and incubated for 10 min on ice. Afterwards, 1 ml of PEG buffer was added, mixed gently by inverting the reaction cups and incubated in a water bath for 10 min at 28 °C. For every transformation plate 250 μl were pipetted to 7.5 ml 40 °C warm overlay media, supplied with uracil and uridine, mixed by inverting and poured over SC medium plates. Next day, the plates were overlaid with SC medium containing FOA.

**Fig. 3:**
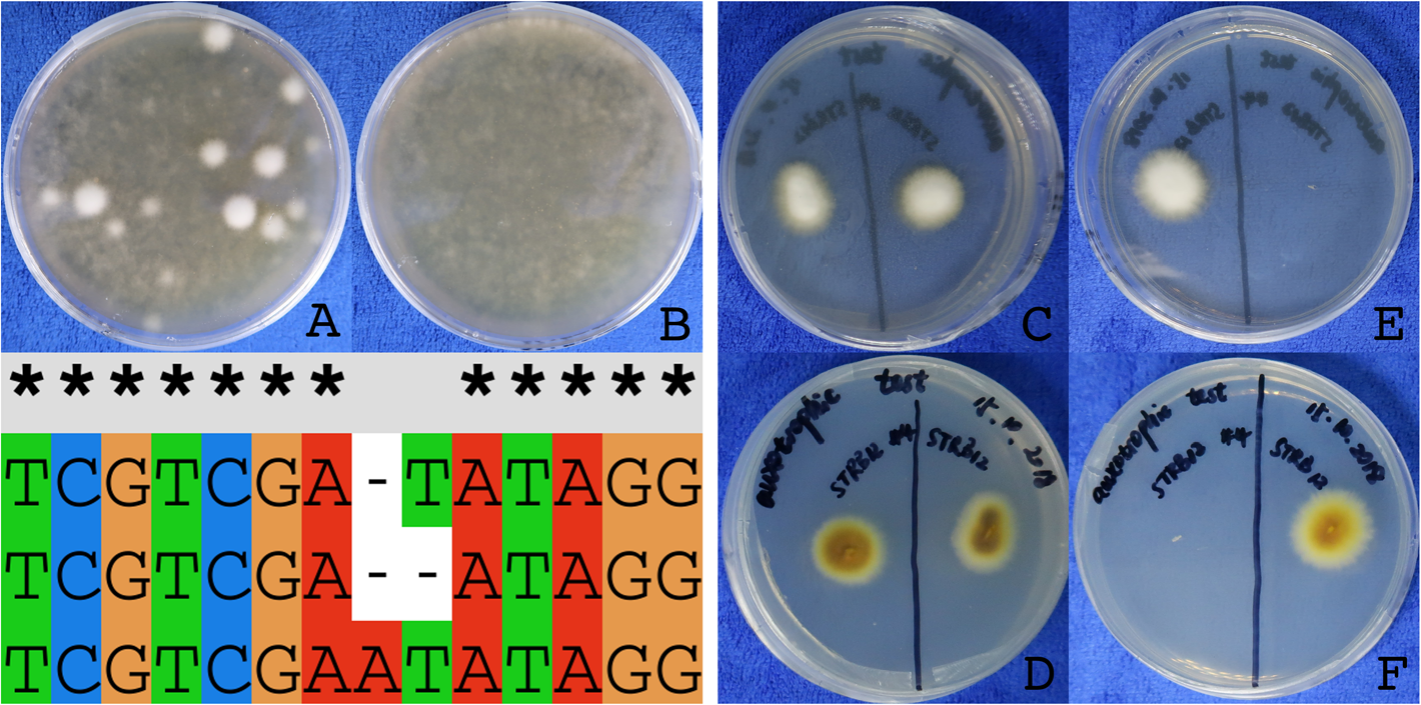
Transformation and analysis of *T. rubrum* ∆ura3 strains. **a** Three weeks old transformation plate supplemented with FOA for counter selection. ∆ura3 strains are visible as distinct colonies. **b** Three weeks old control plate supplemented with FOA for counter selection. **c**, **d** Auxotrophy test of ∆ura3 and parent strain. SC medium supplied with uracil and uridine. The wild-type and the ∆ura3 strain have a similar growth rate **e**, **f**. Auxotrophy test of ∆ura3 and parent strain. SC medium without uracil and uridine. Only the wild-type strain is growing. **g** DNA sequence alignment of the mutated part of the *URA3* gene. Top sequence: Part of the protospacer sequence including PAM sequence. Middle: Auxotrophic strain STRB018. Bottom: Auxotrophic strain STRB019

After two weeks of incubation at 28 °C, distinct colonies have formed on the transformation plates (Fig. 3a), whereas on the control plates only downy hyphae were visible (Fig. 3b). Hyphal fragments of 39 distinct colonies were inoculated to FOA plates and incubated for 3 weeks. All colonies were grown well and hyphal fragments of the border of the colonies were inoculated on SC -uracil selection plates.

After two weeks of inoculation, no growth of the selected strains was observed on -uracil medium, whereas the parent strain, STRB012, was grown also in the absence of uracil. On SC medium, supplemented with uracil and uridine, the transformed strains had a similar growth rate and appearance as the parent strain (Fig. 3c-f).

Three strains were selected for the verification of the genotype and the target regions were sequenced. In each of the three strains the *URA3* gene was mutated 3 to 4 bases upstream of the PAM sequence (Fig. 3g). One strains had a deletion of one nucleotide, one strain had an insertion of one nucleotide and the third had an insertion of 68 nucleotides. The surrounding regions up and downstream had no additional mutations.

Single clone colonies of the two strains with one base indels were isolated and preserved as STRB018 and STRB019 at − 80 °C. The transformation experiment was repeated and resulted a similar amount of transformed strains. Of strain STRB008, which is characterized by a lower amount of microconidia in comparison to STRB012, one uracil auxotrophic mutation had been created and a single clone was preserved as STRB020.

### Target specific knock-out of the *TRP3* gene

**Fig. 4:**
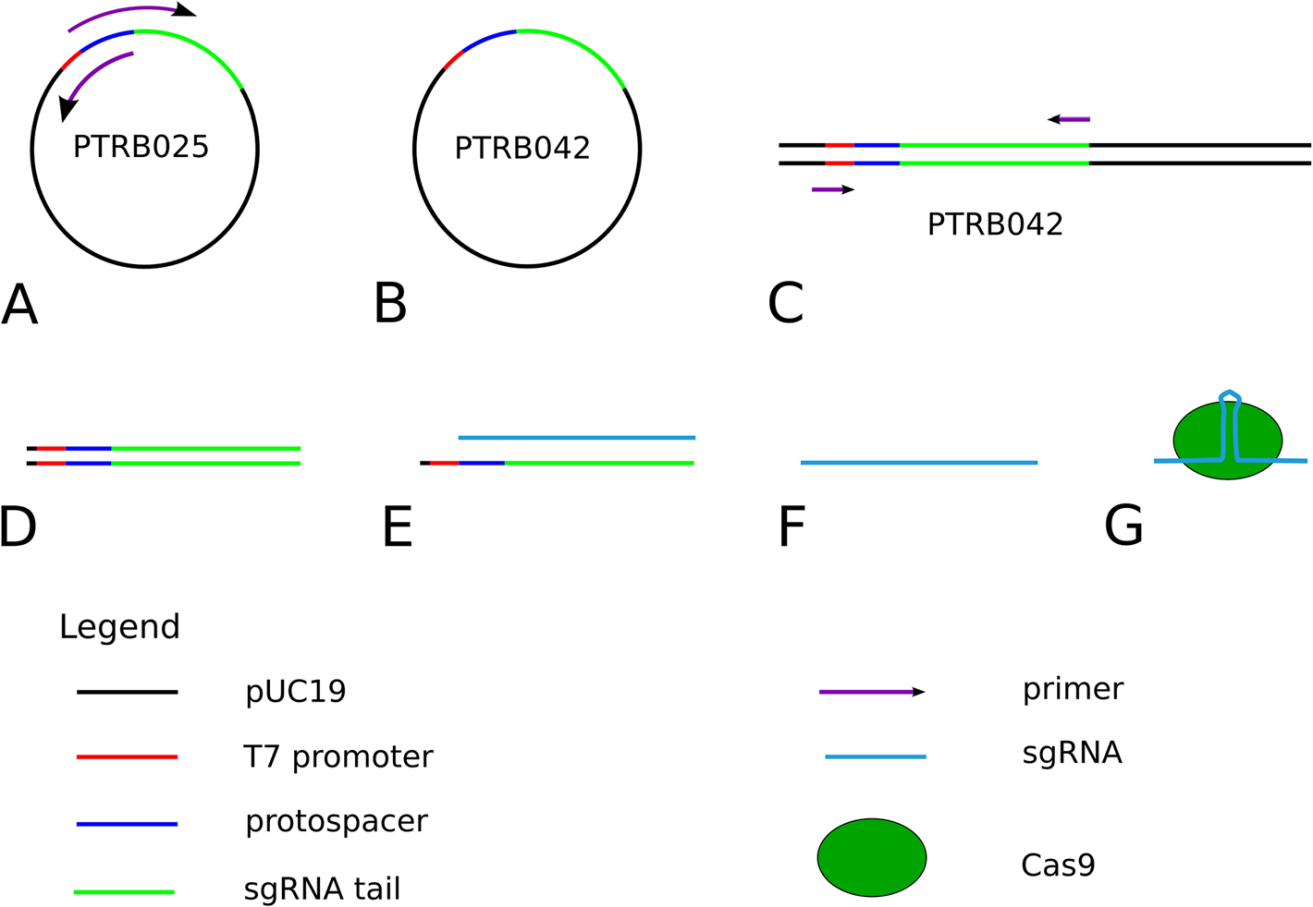
Creation of *TRP3* specific Cas9-sgRNA ribonucleoprotein complex. **a** Amplifying the plasmid PTRB025 with primer pair NTRB107/108 to change the protospacer region. Recirculate the DNA fragment with a Mutagenesis Kit. **b** Amplification of the modified plasmid in *E. coli* and verification of the modification by sequencing with M13 primers. **c** Linearize of the plasmid with HindIII. **d**. Amplification of the sgRNA template with primer pair NTRB80/81. **e** Transcription of the sgRNA with T7 RNA polymerase. **f** DNAse treatment and RNA purification. **g** Cas9-sgRNA ribonucleoprotein complex formation

**Fig. 5:**
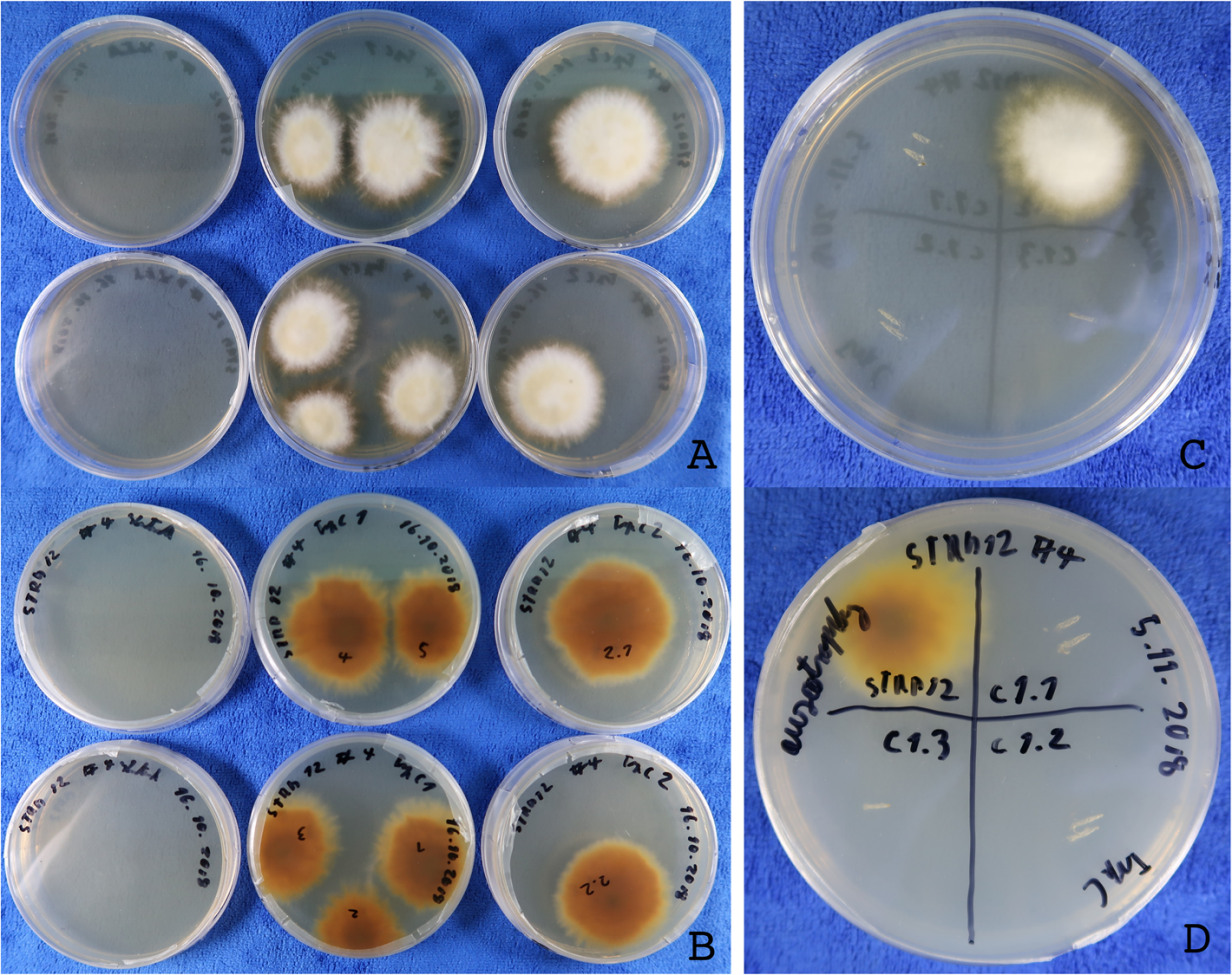
Transformation and analysis of *T. rubrum* ∆trp3. **a** and **b** One month old transformation plates. Control plates (left) without colonies, transformation with sgRNA construct 1 (middle) and transformation with sgRNA construct 2 (right). **c** and **d**. Auxotrophy test of strain 1–3 of construct 1 on SC medium without tryptophan. The wild-type strain STRB012 grown well whereas ∆trp3 strains c1.1-c1.3 are auxotrophic for tryptophan

Next, we were using the ∆*ura3* strain STRB018 for creating tryptophan auxotrophic strains. We predicted TERG07826 as orthologous to the *S. cerevisiae TRP3* gene and designed the primers and vectors. For knocking out the *TRP3* gene, we modified the protocol. First, for the transformation and selection, we used SC medium without uracil. Second, the sgRNA has been transcribed from plasmid PTRB042 resp. 43 (Fig. 4). Further, 3 μg *URA3* fragments has been added to the transformation solution. A total eight colonies appeared on the transformation plates (Fig. 5a, b). On the control plates, without adding Cas9-sgRNA ribonucleoprotein and the *URA3* fragment, no colonies appeared even after one month of cultivation. All eight colonies were inoculated on SC plates without tryptophan and no growth had been observed, even after one month of inoculation (Fig. 5c, d). Both the wild-type strain STRB012 and the ∆*ura3* strain STRB018, were growing on tryptophan lacking media.

Next, we amplified the *Trp3* gene including the flanking region of all strains with the primer pair NTRB076/77 and got in six of the eight samples a single band (Table 1). We cloned four of DNA fragments into pUC19 plasmid for sequencing with the primers NTRB115 and 116. The inserted DNA fragments were in all cases at the predicted position 3–4 base pair upstream of the PAM sequence and in the flanking regions no further mutation occurred. In three of the four strains, the *Ura3* DNA fragments were inserted into the Cas9 DSB sites. The fragment length of the original *Ura3* PCR product was 2964 bp, but all inserted fragments were truncated. In one descendant the inserted fragment had lost one base at each of the ends. The lengths of the inserted *Ura3* fragment of the two other sequenced strains were 2380 resp.1955 bp. In one strain an additional nucleotide had been inserted between the inserted *Ura3* fragment and the theoretical Cas9 blunt end restriction site. Besides the mutations at the cloning site, neither the inserted fragments nor the flanking regions had further mutations.

**Table 1:** Phenotype and length of the inserted marker fragment in the eight ∆trp3 strains

Strain	Phenotype*^1^		Fragment length*^2^		
	Ura	Trp	PCR*^3^	Sequenced*^4^	
STRB012	P	P	NA		NA
STRB018	A	P	NA		NA
∆trp3_c1_1	P	A	2130 bp	+	1955 bp
∆trp3_c1_2	P	A	270 bp	+	0 bp*^5^
∆trp3_c1_3	P	A	2880 bp	+	2962 bp
∆trp3_c1_4	P	A	0 bp	–	NA
∆trp3_c1_5	P	A	2370 bp	+	2380 bp
∆trp3_c2_1	P	A	1900 bp	–	NA
∆trp3_c2_2	P	A	990 bp	–	NA
∆trp3_c2_3	P	A	2250 bp	–	NA

*^1^ A: Auxotrophic, P: Prototrophic

*^2^ length of the inserted *ura3* marker fragment

*^3^ calculated from agarose gel electrophoresis with the LabImage Version 2.62 software. The last digit of the values were rounded up

*^4^ counted from the Sanger sequencing result

*^5^ no *ura3* fragment was inserted, but an unintentional bacterial DNA fragment

## Discussion

We have set up a transformation system for the obligate human pathogenic fungus *T. rubrum*. For this, we had isolated the strain STRB012, which is suitable for routine genetic transformations. Since the transformation protocol is based on creating protoplast from microconidia, we had chosen a strain with a high rate of conidiogenesis. As in other *T. rubrum* strains, the conidiogenesis can even be enhanced by increasing the CO_2_ concentration in the atmosphere [9]. To prevent genetic changes by serial transfer, the procedure from isolation, via monoclonal isolation until freeze-stock has been kept as short as possible [10]. Moreover, to ensure homogeneous genetic material, the freeze-stock had been aliquot. So far, the selection of transformed *Trichophyton* strains was based on drug resistances genes, mostly the hygromycin B resistances gene. Thereby, most of the genetic manipulations in the genus *Trichophyton* were performed with hygromycin B susceptible *T. mentagrophytes* strains. For example, the growth of *T. mentagrophytes* CBS 570.80 was completely inhibited at a concentration of 20 μg/ml and selected at a final concentration of 100 μg/ml hygromycin B [11]. Mutations of *T. mentagrophytes* TIMM2789 were selected with a concentration of 250 μg/ml hygromycin B in the overlay medium [12]. On the other hand, *T. rubrum* CBS118892 has a 10 times higher tolerance for hygromycin B with a minimum inhibitory concentration (MIC) of 200 μg/ml for microconidia and 800 μg/ml for arthroconidia [13]. Besides this, the selection with auxotrophic markers has the advantage of being more stringent and ∆ura3 strain do not grow in the absence of uracil.

For creation of uracil auxotrophic strains, we first predicted, which gene in the genome of *T. rubrum* could be homologous to the *Ura3* gene of *S. cerevisiae*. By knocking out this gene, we resulted uracil auxotrophic strains and used for the counter selection 1 g/l FOA. Whereas this concentration in yeast is sufficient, the *T. rubrum* wild-type exhibited slow growth and had been visible as downy hyphae. Since the transformed strains were visible as distinct colonies, they were isolated by inoculating to fresh FOA plates. To proof undoubtfully, that the mutation in the *URA3* gene is the cause of auxotrophy, we recovered the function by knock in the *URA3* gene. For this, we chose the *TRP3* gene as a target and changed the genotype from *∆URA3, TRP3* into ∆TRP3, *URA3*.

We have demonstrated that gene knock-outs in *T. rubrum* can be created with Cas9 in two ways. First, by inducing DSBs with a Cas9 ribonucleoprotein complex. In the process from inducing the DSB until the NHEJ repair, with a certain likelihood, mutations occurs. In *S. cerevisiae* a significant fraction of the mutations were + 1 indels caused by 1 bp 5′ overhangs at the Cas9 cleavage sites. Thereby most of the + 1 indels required the DNA polymerase Pol4 to fill in 5′ overhangs [14]. These + 1 indels are causing frame shifts in the gene translation and are disabling the protein function. Second, by inducing DSBs and integration of a selection marker. Since the *URA3* selection marker has several stop codons in all 6 reading frames, the translation will be interrupted and the knock-out strains could be selected by using the uracil selection. In both cases the DSBs had been repaired by NHEJ.

In *Aspergillus nidulans* both NHEJ and HDR pathways are engaged in repairing Cas9 induced DNA DSBs. A NHEJ proficient *A. nidulans* strain could be transformed with Cas9 without supplying homologous DNA fragments, whereas NHEJ deficient strains required gene-targeting substrates (GTS) for efficient transformation. The GTSs had homologous sequences to both flanking sites of the DSBs whereby the highest efficiencies had circular GTSs with 1000 - 2000 bp homologous sequences. Interestingly, also single-stranded DNA fragments with a length of 90 bp induced the HDR mechanism [15]. Whereas in *Trichophyton* and *Aspergillus* spp. the NHEJ is dominant, in *S. cerevisiae* quasi all DSBs are repaired by HDR in the presence of a homologous template [16]. Also in *Candida glabrata* only a modest increase in targeting efficiency (1.5% vs 1.1%) was seen in NHEJ deficient strain compared to the wild-type [17].

Genetic manipulations of *S. cerevisiae* are now performed for more than 40 years and many tools and protocols have been developed in this time. Nowadays, genetic manipulations of *S. cerevisiae,* and as well of *C. albicans*, are routines, whereas transformations of non-model fungal species are still often challenging. We developed a transformation protocol for such a species and are convinced, that the protocol can be adapted to other fungal species. With the development of NGS and bioinformatics tools, it is now possible to predict the *URA3* gene sequence of nearly every fungal strain with reasonable effort. The developed protocol is not depending on further species specific tools as autonomous replication vectors. We are convinced that with further developments of the NGS in combination with the Cas9 technique the knowledge of the genetics of non-model fungal species will increase significantly in the near future.

## Conclusions

In fungal model organism, gene knock-out are routinely performed by using an integrating selection marker, which allows selection against the auxotrophic parent strain, into the target gene. We developed for the first time a protocol, based on in vitro synthesized Cas9-sgRNA ribonucleoprotein complex, to adapt the well established method to non-model fungal species. We knocked out the *Ura3* gene in *T. rubrum* and used this strain for target specific gene knock-outs. The gene knock-out of the *Ura3* gene as well of the *Trp3* gene led to auxotrophy in *T. rubrum*.

## Materials and methods

### Fungal strains

The fungal strains STRB008 and STRB012 were isolated at the Institute of Dermatology in Nanjing, China. Both strains were isolated by the clinical personal of the hospital for diagnosis and treatment purpose. Strain STRB008 was isolated in June 2017 from a foot nail and strain STRB012 was isolated in July 2017 from the leg of a patient. All created knock-out strains were derived from clinical isolate STRB012 except of the strain STRB020, which was a descendant of clinical isolate STRB008 (Table 2).

**Table 2:** *T. rubrum* strains

Strain	Parent strain	Phenotype	Genotype
STRB008^*^		WT	WT
STRB012^*^		WT	WT
STRB018^*^	STRB012	Ura^−^	ura3-∆363^**^
STRB019^*^	STRB012	Ura^−^	ura3-363_364insA^**^
STRB020^*^	STRB008	Ura^−^	
∆Trp_c1.1	STRB018	Trp^−^	ura3-∆363, trp3::URA3^**^
∆Trp_c1.2	STRB018	Trp^−^	ura3-∆363, trp3::URA3^**^

*preserved at the Chinese National Medical Fungal Collection in Nanjing

**verified by sequencing

### PCR and vector

PCR reactions were performed in 25 or 50 μl using Pfu polymerase (Vazyme, China, Cat#P505) with an initial denaturation step at 95 °C for 2 min, 30 to 35 cycles of denaturation for 45 s, annealing at 50–60 °C for 30s and elongation at 72 °C for 1 min per 1 kb. The finalization at 72 °C had a duration of 1–2 times of the elongation. Vectors (Table 3) were constructed by using the pUC19 backbone and manipulated with the Mutagenesis Kit (Vazyme, #C215) or by sticky end restriction and T4 DNA ligation. Plasmids were amplified in *E. coli* DH5α and purified using a Plasmid isolation kit (Qiagen, Germany, #27106).

**Table 3:** Plasmids

Plasmid^*^	Insert
PTRB024	orotidine 5-phosphate decarboxylase (‘Ura3’) with flanking regions
PTRB025	template plasmid to synthesis Ura3 sgRNA
PTRB035	anthranilate synthase component 2 (‘Trp3)’ with flanking regions
PTRB042	template plasmid to synthesis Trp3 sgRNA
PTRB043	template plasmid to synthesis Trp3 sgRNA

^*^The complete sequences of the plasmids, including annotation, are given in the Additional file 1

For the construction of the plasmid PTRB024, the *URA3* gene had been amplified from strain STRB012 with the primer pair NTRB060/61 and restricted with EcoRI/BamHI. The cloning vector had been restricted and dephosphorylated before inserting the *URA3* fragment.

We constructed a vector, PTRB025, containing the sgRNA fragment of pFC334 (Addgene, USA, #87846) [18], the T7 promoter and a protospacer unique for the *T. rubrum URA3* gene. Afterwards, the fragment was amplified with the primer pair NTRB80/81, agarose gel extracted and used as template for the sgRNA transcription. The transcription had been performed with a T7 RNA polymerase (Vazyme, #TR101) according to the producer manual. We used 500 ng of the template DNA and incubated for 4 h at 37 °C. Afterwards, the transcript was purified with a RNA Clean-Up Kit (Takara, Japan, #632638).

After purification, 4 μg sgRNA was mixed with 12 μg Cas9 protein (Takara, #632641) in 30 μl TE buffer and incubated for 5 min at 37 °C to induced complex formation. After adding 5 μl of a 50 mMol spermidine (Sigma-Aldrich, USA, #S0266) solution, the transformation solution was stored at − 80 °C until use. The plasmid PTRB042 and 43 were constructed by exchanging the protospacer region of PTR025 from *URA3* to *TRP3* homologous by using the primer pairs NTRB107/108 resp. 109/110. The sgRNA was transcribed from linearized DNA fragments of PTRB042 (‘construct 1’) and 43 (‘construct 2’). For the Uracil selection, 3 μg *URA3* fragments with flanking regions were amplified from plasmid PTRB024 with the primer pair NTRB85/86.

### Transformation

For the transformation, KCl buffer (1 M KCl in 10 mMol sodium phosphate buffer, pH 5.8), lysis buffer (KCl buffer with 10 mg/ml lysing enzymes (Sigma-Aldrich, #L1412) and driselase (Sigma-Aldrich, #D9515); stored at − 20 °C), sucrose buffer (1 M sucrose, 10 mMol Tris HCl pH 7.5, 10 mMol CaCl_2_) and a PEG buffer (60% (w/v) PEG 4000, 10 mMol Tris HCl pH 7.5, 10 mMol CaCl_2_) were prepared.

The overlay media were based on SC medium (see Additional file 2) supplemented with 1 M sucrose and 1% low melting agarose (Sigma-Aldrich, #A9045). The media were melted at 80 °C and cooled down to 40 °C before adding the protoplasts. For the FOA counter-selection, the media have been adjusted to pH 5 and 1 g/l FOA (Zymo Research, USA, #F9001) were added.

### Sequencing

For Next Generation Sequencing (NGS), 0.5 g mycel from 10 days old liquid cultures were harvested and disrupted in liquid nitrogen. DNA was extracted using Qiagen plant extraction kit. The fragments, approximate 400 bp length, were pair-end sequenced with read length of 150 bp using the Hiseq 4000 platform. The sequenced fragments were mapped with Bowtie 2 [19] and aligned with Samtools [20] to the reference genome *T. rubrum* CBS 118892 (ASM15142v1). The alignments of the Fastq files to the reference assembly was visualized with Consed [21] and the regions of interest were analyzed for polymorphisms before cloning and primer designing. The ITS1 and 2 sequences were extracted from the NGS data and aligned with ClustalX.

## Supplementary information

**Additional file 1.** Supplement_sequences; DNA sequences of plasmids, primers and ITS 1 and 2 including alignment.

**Additional file 2.** Supplement_media; cultivation media.

## Data Availability

The datasets used and/or analysed during the current study are available from the corresponding author on reasonable request.

## Abbreviations

Cas9: Protein of the CRISPR/Cas9 system
DSB: Double string break in a chromosome
FOA: 5-fluoroorotic acid
HDR: Homologous directed repair of a chromosome
NHEJ: Non-homologous end joining of a chromosome
NTRB0XX: Nucleic acid and primers
PTRB0XX: Plasmids
SC: Synthetic Complete medium
sgRNA: Small guided RNA of the Cas9-sgRNA ribonucleoprotein complex
STRB0XX: *T. rubrum* strain
Tr1: Trichophyton cultivation medium Nr. 1
*TRP3*: *Anthranilate synthase component 2 gene*
URA3: Orotidine-5′-monophosphate decarboxylase gene

## References

[1] Alshahni MM, Yamada T (2017). Genetic manipulations in Dermatophytes. Mycopathologia.

[2] Boeke Jef D., La Croute Francois, Fink Gerald R. (1984). A positive selection for mutants lacking orotidine-5′-phosphate decarboxylase activity in yeast: 5-fluoro-orotic acid resistance. Molecular and General Genetics MGG.

[3] Kaufman G, Horwitz BA, Hadar R, Ullmann Y, Berdicevsky I (2004). Green fluorescent protein (GFP) as a vital marker for pathogenic development of the dermatophyte *Trichophyton mentagrophytes*. Microbiology.

[4] Yamada T, Makimura K, Hisajima T, Ishihara Y, Umeda Y, Abe S (2009). Enhanced gene replacements in *Ku80* disruption mutants of the dermatophyte, *Trichophyton mentagrophytes*. FEMS Microbiol Lett.

[5] Wu WY, Lebbink JHG, Kanaar R, Geijsen N, van der Oost J (2018). Genome editing by natural and engineered CRISPR-associated nucleases. Nat Chem Biol.

[6] Zhang C, Meng X, Wei X, Lu L (2016). Highly efficient CRISPR mutagenesis by microhomology-mediated end joining in *Aspergillus fumigatus*. Fungal Genet Biol.

[7] Nagy G, Szebenyi C, Csernetics A, Vaz AG, Tóth EJ, Vágvölgyi C, Papp T (2017). Development of a plasmid free CRISPR-Cas9 system for the genetic modification of *Mucor circinelloides*. Sci Rep.

[8] Wang Q, Cobine PA, Coleman JJ (2018). Efficient genome editing in *Fusarium oxysporum* based on CRISPR/Cas9 ribonucleoprotein complexes. Fungal Genet Biol.

[9] Laurent A, Monod M (2017). Production of *Trichophyton rubrum* microspores in large quantities and its application to evaluate amorolfine/azole compound interactions in vitro. Mycoses.

[10] Homolka L (2014). Preservation of live cultures of basidiomycetes - recent methods. Fungal Biol.

[11] Gonzalez R, Ferrer S, Buesa J, Ramon D (1989). Transformation of the dermatophyte *Trichophyton mentagrophytes* to hygromycin B resistance. Infect Immun.

[12] Yamada T, Makimura K, Uchida K, Yamaguchi H (2005). Reproducible genetic transformation system for two dermatophytes, *Microsporum canis* and *Trichophyton mentagrophytes*. Med Mycol.

[13] Coelho LM, Aquino-Ferreira R, Maffei CM, Martinez-Rossi NM (2008). In vitro antifungal drug susceptibilities of dermatophytes microconidia and arthroconidia. J Antimicrob Chemother.

[14] Lemos BR, Kaplan AC, Bae JE, Ferrazzoli AE, Kuo J, Anand RP, Waterman DP, Haber JE (2018). CRISPR/Cas9 cleavages in budding yeast reveal templated insertions and strand-specific insertion/deletion profiles. Proc Natl Acad Sci U S A.

[15] Nødvig CS, Hoof JB, Kogle ME, Jarczynska ZD, Lehmbeck J, Klitgaard DK, Mortensen UH (2018). Efficient oligo nucleotide mediated CRISPR-Cas9 gene editing in Aspergilli. Fungal Genet Biol.

[16] Hinnen A, Hicks JB, Fink GR (1978). Transformation of yeast. Proc Natl Acad Sci U S A.

[17] Cen Y, Timmermans B, Souffriau B, Thevelein JM, Van Dijck P (2017). Comparison of genome engineering using the CRISPR-Cas9 system in *C. glabrata* wild-type and *lig4* strains. Fungal Genet Biol.

[18] Nødvig CS, Nielsen JB, Kogle ME, Mortensen UH (2015). A CRISPR-Cas9 system for genetic engineering of filamentous Fungi. PLoS One.

[19] Langmead B, Salzberg S (2012). Fast gapped-read alignment with bowtie 2. Nat Methods.

[20] Li H, Handsaker B, Wysoker A, Fennell T, Ruan J, Homer N, Marth G, Abecasis G, Durbin R (2009). 1000 genome project data processing subgroup. The sequence alignment/map (SAM) format and SAMtools. Bioinformatics.

[21] Gordon D, Green P (2013). Consed: a graphical editor for next-generation sequencing. Bioinformatics.

