# Supplementary material for: Auxotrophic mutations of Trichophyton rubrum created by in vitro synthesized Cas9 ribonucleoprotein
Source: BMC Biotechnol. 2020 Jan 20;20:6. doi: 10.1186/s12896-020-0601-z (PMC6971929; doi:10.1186/s12896-020-0601-z)
Supplement: Supplementary file 1 — Additional file 1. Supplement_sequences; DNA sequences of plasmids, primers and ITS 1 and 2 including alignment. [file 12896_2020_601_MOESM1_ESM.pdf]

>PTRB024 *Trichophyton rubrum* 'STRB012' orotidine 5-phosphate decarboxylase with flanking regions; brown: pUC19, green: *Ura3*, blue: protospacer homologous, underline: primer homologous

TCGCGCGTTTCGGTGATGACGGTGAAAACCTCTGACACATGCAGCTCCCGGAGACGGTCACAGCTTGTCTGTAAGCGGATGC
CGGGAGCAGACAAGCCCGTCAGGGCGCGTCAGCGGGTGTTGGCGGGTGTCGGGGCTGGCTTAACTATGCGGCATCAGAGCAG
ATTGTACTGAGAGTGCACCATATGCGGTGTGAAATACCGCACAGATGCGTAAGGAGAAAATACCGCATCAGGCGCCATTCGC
CATTCAGGCTGCGCAACTGTTGGGAAGGGCGATCGGTGCGGGCCTCTTCGCTATTACGCCAGCTGGCGAAAGGGGGATGTGC
TGCAAGGCGATTAAGTTGGGTAACGCCAGGGTTTTCCCAGTCACGACGTTGTAAAACGACGGCCAGTGAATTCCAGTTAACA
TTTGTTTGACTCGAAATGCCTTTTATTAATACATCCTGTCCGGGCTGTTTAGATGCTTGCCCGAATGAGAGCAATTCCGGCC
TTTTCATTGTCCCTTCCACTGTTCCTCGACATCACCATGCTCAATGATACAGGCAGCGATGCGTTGACAGTCTTCGACACAG
ACCTAATCGCGTTAGGCATCGCTCCAACGTACCTGGGTTTTGGGCCTCAAACTCAAGCTTGACAGCTAATGGGATTGTTTTA
CGGCAAGTTATCTACGTTGAGATCCAGCTACTTGATTCACAACGTAATCCTATTTCAGATTGGATTCTCGAGGAATCTGTCG
TTGTTCCGTCAGCTGAAGGTAACACTAGGCTGTCCGGCAGAGGGATGAGGGATTGTCTGTATTTTGCAACAGCACCAGGTAA
CCAACAGCTATACGTGGCCGAAAAGAAGAATGGCATTGTACAGCAGCTTCCTGTTGTTTAATTGCCTTGAATCATTACTTTA
CCGGAATGATGACCATGATTCTATTTGATACACTTTATTAACTATTAACTACCGCTACAAATGAAGAGGCATCCCTTTGCTC
TTGTGTCCCAAATAGTTGCCCTGCACCGCAACCAGCGAGATAGTTCTTCCTTCAGATACCATCAACATGCTTCTACAACGAT
GTAGGGTGTAAAAAGAAATATCACCAGAACAGTGAGATAGATATATATATACCATATTTAGAGCGAAAAAAAAAAGAACGGG
GCCCTGCCGCGACTCGAACGCGGGGCCTCTCGCATCTTTGGTGTCCCAAAGCGAGAATCATACCACTAGACCACAAGGCCAT
TCTTGTCAGCAGAGAGACAGGGGGTTGCTCATAGTGATAAGCCATCAGAGTGCAGCAATATTTCTCCTGGTGTCTACCACTT
ATTACGTATCATTTGATCGATGTTAATGCCAGCTCGACAGCTCAACGTTTCACCTTTTTGTAGTTCAAAGAAAGAGACTACT
TTTTTTTTCGTGTGCGAGATAGGAGAACCAAGGAAAATGTCTTAGTCAGATGAATAGCTGACGTTACACAGATCACGTTATG
TGATAAAAGAATTGACCAATAAGACAGCAGTAACAAATAAGCTTAATCGTCATTAGTCTTGGCACTGGCTATATCGACGACG
CCTTGCCCTGTGCGGGACATCTAAATATCTACTTCCAGGTCCAGCCCGGGCTCTCGAGGGACAAACGACAAAGGCACGATGG
CGTCGAGATCTCAGGTCTCGTACGGGGACCGTGCAAAGACACACCCGAACCCCCTTGTGCGGCGGCTGTTCGAGATCGCAGA
GCAGAAACAGTCCAACGTCGTCGTCTCCGCAGATGTCACCACCACGAGCGCATTGCTAGACCTGGCCGAGCGTGAGTCTTGA
TACTTTATGAAGAGAAGAAGGAGAAGAAGCAATAGCTGACAATAAGGACACAAGACAGGCCTCGGACCGCACATGGTCGTAC
TCAAGACACACATCGACATCATCACTGATTTCTCCCCGCAGACAGTCGAGGGCCTCAAGGCCGCCGCTCAGAAACACAACTT
CCTCATCTTCGAGGACAGAAAGTTCGTCGATATAGGCCACACAGTCCAGATGCAGTACCATGGTGGCAGCCTACGCATCTCC
GAATGGGCGCATATCGTCAACTGTGCCGTGCTAGCGGGGTCAGGCATTGTCGACGCTCTAGCGCAGATATCTTCCTCGGGCG
TATTCCCTTACCCCGCTGGCGAGAGGGGACTGTTGATTCTCGCCGAGATGACTTCCAAGGGCTCATTGGCAACGGGCGAGTA
TACGAAGCTGTCGGTTGAGATGGCCAGGAGACATGCTGGGTTCGTCATGGGGTTCGTGGCTACGCGGTCCCTGGGCGATATC
GAGACAGAGACTGAGCGCAAGGCCGATGAAGACTTTGTGCTATTCACCACGGGTGTCAATCTTGCCTCGAAGGGTGACCAGT
TAGGCCAGCAGTATCAGACTCCCGAGTCGGCTGTGGGCAGAGGGGCGGACTTTATCATCTCGGGGAGAGGTATCTATGCTGC
TCCTGACCCTGTGGAGGCCATAAAGCAGTACCAATCTGCCGGCTGGAGTGCATACCTCAAGCGTATATCCAGATAATTAAAA
CGAAAGAAAGAAAGGAAAAAGTTCATGCTTAAGATCCCACCAATAGCTTCATTTATACCTTATAACTAGAAAGAAAAGAAAG
CAGAATATCATACATAATCGTAGGCTAGAATGGGGAAGCAGGAGCTTGCATCATGGGCAATGCGACGCCAAACACCGTCATG
CCCCAGAGCGCCATCTGAGCGAACCAGAAGCCGCCACCGTACTTCCGTCCGGCTCCCGCAGCCTGCCCGGAGTAGACATATC
CGTACAAGAACAGCACGCGCATCACCACCCATGTAGCTCCGATCAACGTCGTGGCGGCAGGATACTTGAGTCCAGCAACGAG
AGTGAGCAGCATCGTCTGTGGTGCGTTCTCAAGGTAGTTCGTGTGGGCTCGCTGGGCGCAGTTGAACGCATCGGCTTTGGGC
TGTGGGGGAGGCCAATTGGTTAGCAGTTGAATCAAGCTGCACAGTTGTACACACTTGCATTAAGGGAAGACTCACGTCGGCC
TTGCATTGTTCGATAGTGGCATAGCTGTGAGGATAGGGGATGCCGGCAGCTCTGCGCTTGATGCCAGTGATGATGCCGTGGA
CGAAGCCGAGCACTGGGATGGCGCCGAGGGCGACGGCGATGATGTTGCTGGCGGGCAGATGTCAAGTTAGCTGTGCTCTCCC
TGGATCCTCTAGAGTCGACCTGCAGGCATGCAAGCTTGGCGTAATCATGGTCATAGCTGTTTCCTGTGTGAAATTGTTATCC
GCTCACAATTCCACACAACATACGAGCCGGAAGCATAAAGTGTAAAGCCTGGGGTGCCTAATGAGTGAGCTAACTCACATTA
ATTGCGTTGCGCTCACTGCCCGCTTTCCAGTCGGGAAACCTGTCGTGCCAGCTGCATTAATGAATCGGCCAACGCGCGGGGA
GAGGCGGTTTGCGTATTGGGCGCTCTTCCGCTTCCTCGCTCACTGACTCGCTGCGCTCGGTCGTTCGGCTGCGGCGAGCGGT
ATCAGCTCACTCAAAGGCGGTAATACGGTTATCCACAGAATCAGGGGATAACGCAGGAAAGAACATGTGAGCAAAAGGCCAG
CAAAAGGCCAGGAACCGTAAAAAGGCCGCGTTGCTGGCGTTTTTCCATAGGCTCCGCCCCCCTGACGAGCATCACAAAAATC
GACGCTCAAGTCAGAGGTGGCGAAACCCGACAGGACTATAAAGATACCAGGCGTTTCCCCCTGGAAGCTCCCTCGTGCGCTC
TCCTGTTCCGACCCTGCCGCTTACCGGATACCTGTCCGCCTTTCTCCCTTCGGGAAGCGTGGCGCTTTCTCATAGCTCACGC
TGTAGGTATCTCAGTTCGGTGTAGGTCGTTCGCTCCAAGCTGGGCTGTGTGCACGAACCCCCCGTTCAGCCCGACCGCTGCG
CCTTATCCGGTAACTATCGTCTTGAGTCCAACCCGGTAAGACACGACTTATCGCCACTGGCAGCAGCCACTGGTAACAGGAT
TAGCAGAGCGAGGTATGTAGGCGGTGCTACAGAGTTCTTGAAGTGGTGGCCTAACTACGGCTACACTAGAAGAACAGTATTT
GGTATCTGCGCTCTGCTGAAGCCAGTTACCTTCGGAAAAAGAGTTGGTAGCTCTTGATCCGGCAAACAAACCACCGCTGGTA
GCGGTGGTTTTTTTGTTTGCAAGCAGCAGATTACGCGCAGAAAAAAAGGATCTCAAGAAGATCCTTTGATCTTTTCTACGGG
GTCTGACGCTCAGTGGAACGAAAACTCACGTTAAGGGATTTTGGTCATGAGATTATCAAAAAGGATCTTCACCTAGATCCTT
TTAAATTAAAAATGAAGTTTTAAATCAATCTAAAGTATATATGAGTAAACTTGGTCTGACAGTTACCAATGCTTAATCAGTG
AGGCACCTATCTCAGCGATCTGTCTATTTCGTTCATCCATAGTTGCCTGACTCCCCGTCGTGTAGATAACTACGATACGGGA

GGGCTTACCATCTGGCCCCAGTGCTGCAATGATACCGCGAGACCCACGCTCACCGGCTCCAGATTTATCAGCAATAAACCAG
CCAGCCGGAAGGGCCGAGCGCAGAAGTGGTCCTGCAACTTTATCCGCCTCCATCCAGTCTATTAATTGTTGCCGGGAAGCTA
GAGTAAGTAGTTCGCCAGTTAATAGTTTGCGCAACGTTGTTGCCATTGCTACAGGCATCGTGGTGTCACGCTCGTCGTTTGG
TATGGCTTCATTCAGCTCCGGTTCCCAACGATCAAGGCGAGTTACATGATCCCCCATGTTGTGCAAAAAAGCGGTTAGCTCC
TTCGGTCCTCCGATCGTTGTCAGAAGTAAGTTGGCCGCAGTGTTATCACTCATGGTTATGGCAGCACTGCATAATTCTCTTA
CTGTCATGCCATCCGTAAGATGCTTTTCTGTGACTGGTGAGTACTCAACCAAGTCATTCTGAGAATAGTGTATGCGGCGACC
GAGTTGCTCTTGCCCGGCGTCAATACGGGATAATACCGCGCCACATAGCAGAACTTTAAAAGTGCTCATCATTGGAAAACGT
TCTTCGGGGCGAAAACTCTCAAGGATCTTACCGCTGTTGAGATCCAGTTCGATGTAACCCACTCGTGCACCCAACTGATCTT
CAGCATCTTTTACTTTCACCAGCGTTTCTGGGTGAGCAAAAACAGGAAGGCAAAATGCCGCAAAAAAGGGAATAAGGGCGAC
ACGGAAATGTTGAATACTCATACTCTTCCTTTTTCAATATTATTGAAGCATTTATCAGGGTTATTGTCTCATGAGCGGATAC
ATATTTGAATGTATTTAGAAAAATAAACAAATAGGGGTTCCGCGCACATTTCCCCGAAAAGTGCCACCTGACGTCTAAGAAA
CCATTATTATCATGACATTAACCTATAAAAATAGGCGTATCACGAGGCCCTTTCGTC

>PTRB025 *Trichophyton rubrum Ura3* sgRNA with T7 promoter; brown: pUC19, red: T7 promoter, blue: protospacer homologous, green: sgRNA tail, underline: primer homologous
TCGCGCGTTTCGGTGATGACGGTGAAAACCTCTGACACATGCAGCTCCCGGAGACGGTCACAGCTTGTCTGTAAGCGGATGC
CGGGAGCAGACAAGCCCGTCAGGGCGCGTCAGCGGGTGTTGGCGGGTGTCGGGGCTGGCTTAACTATGCGGCATCAGAGCAG
ATTGTACTGAGAGTGCACCATATGCGGTGTGAAATACCGCACAGATGCGTAAGGAGAAAATACCGCATCAGGCGCCATTCGC
CATTCAGGCTGCGCAACTGTTGGGAAGGGCGATCGGTGCGGGCCTCTTCGCTATTACGCCAGCTGGCGAAAGGGGGATGTGC
TGCAAGGCGATTAAGTTGGGTAACGCCAGGGTTTTCCCAGTCACGACGTTGTAAAACGACGGCCAGTGAATTCGAGCTCGGT
ACCCGGGGATCCTCTAGAGTCGACCTGCAGGTAATACGACTCACTATAGGACAGAAAGTTCGTCGATATGTTTTAGAGCTAG
AAATAGCAAGTTAAAATAAGGCTAGTCCGTTATCAACTTGAAAAAGTGGCACCGAGTCGGTGCTTTTGGCCGGCATGGTCCC
AGCCTCCTCGCTGGCGCCGGCTGGGCAACATGCTTCGGCATGGCGAATGGGACTGATTTAATAGCTCCATGTCAACAAGAAT
AAAACGCGTTTCGGGTTTACCTCTTCCAGATACAGCTCATCTGCAATGCATTAATGCATTGGACCTCGCAACCCTAGTACGC
CCTTCAGGCTCCGGCGAAGCAGAAGAATAGCTTAGCAGAGTCTATTTTCATTTTCGGGAGACGAGATCAAGCAGATCAACGG
TCGTCAAGAGACCTACGAGACTGAGGAATCCGCTCTTGGCTCGCGGCCGCCGAGACAGCAGAATCACCGCCCAAGTTAAGCC
TTTGTGCTGATCATGCTCTCGAACGAAGCTTGGCGTAATCATGGTCATAGCTGTTTCCTGTGTGAAATTGTTATCCGCTCAC
AATTCCACACAACATACGAGCCGGAAGCATAAAGTGTAAAGCCTGGGGTGCCTAATGAGTGAGCTAACTCACATTAATTGCG
TTGCGCTCACTGCCCGCTTTCCAGTCGGGAAACCTGTCGTGCCAGCTGCATTAATGAATCGGCCAACGCGCGGGGAGAGGCG
GTTTGCGTATTGGGCGCTCTTCCGCTTCCTCGCTCACTGACTCGCTGCGCTCGGTCGTTCGGCTGCGGCGAGCGGTATCAGC
TCACTCAAAGGCGGTAATACGGTTATCCACAGAATCAGGGGATAACGCAGGAAAGAACATGTGAGCAAAAGGCCAGCAAAAG
GCCAGGAACCGTAAAAAGGCCGCGTTGCTGGCGTTTTTCCATAGGCTCCGCCCCCCTGACGAGCATCACAAAAATCGACGCT
CAAGTCAGAGGTGGCGAAACCCGACAGGACTATAAAGATACCAGGCGTTTCCCCCTGGAAGCTCCCTCGTGCGCTCTCCTGT
TCCGACCCTGCCGCTTACCGGATACCTGTCCGCCTTTCTCCCTTCGGGAAGCGTGGCGCTTTCTCATAGCTCACGCTGTAGG
TATCTCAGTTCGGTGTAGGTCGTTCGCTCCAAGCTGGGCTGTGTGCACGAACCCCCCGTTCAGCCCGACCGCTGCGCCTTAT
CCGGTAACTATCGTCTTGAGTCCAACCCGGTAAGACACGACTTATCGCCACTGGCAGCAGCCACTGGTAACAGGATTAGCAG
AGCGAGGTATGTAGGCGGTGCTACAGAGTTCTTGAAGTGGTGGCCTAACTACGGCTACACTAGAAGAACAGTATTTGGTATC
TGCGCTCTGCTGAAGCCAGTTACCTTCGGAAAAAGAGTTGGTAGCTCTTGATCCGGCAAACAAACCACCGCTGGTAGCGGTG
GTTTTTTTGTTTGCAAGCAGCAGATTACGCGCAGAAAAAAAGGATCTCAAGAAGATCCTTTGATCTTTTCTACGGGGTCTGA
CGCTCAGTGGAACGAAAACTCACGTTAAGGGATTTTGGTCATGAGATTATCAAAAAGGATCTTCACCTAGATCCTTTTAAAT
TAAAAATGAAGTTTTAAATCAATCTAAAGTATATATGAGTAAACTTGGTCTGACAGTTACCAATGCTTAATCAGTGAGGCAC
CTATCTCAGCGATCTGTCTATTTCGTTCATCCATAGTTGCCTGACTCCCCGTCGTGTAGATAACTACGATACGGGAGGGCTT
ACCATCTGGCCCCAGTGCTGCAATGATACCGCGAGACCCACGCTCACCGGCTCCAGATTTATCAGCAATAAACCAGCCAGCC
GGAAGGGCCGAGCGCAGAAGTGGTCCTGCAACTTTATCCGCCTCCATCCAGTCTATTAATTGTTGCCGGGAAGCTAGAGTAA
GTAGTTCGCCAGTTAATAGTTTGCGCAACGTTGTTGCCATTGCTACAGGCATCGTGGTGTCACGCTCGTCGTTTGGTATGGC
TTCATTCAGCTCCGGTTCCCAACGATCAAGGCGAGTTACATGATCCCCCATGTTGTGCAAAAAAGCGGTTAGCTCCTTCGGT
CCTCCGATCGTTGTCAGAAGTAAGTTGGCCGCAGTGTTATCACTCATGGTTATGGCAGCACTGCATAATTCTCTTACTGTCA
TGCCATCCGTAAGATGCTTTTCTGTGACTGGTGAGTACTCAACCAAGTCATTCTGAGAATAGTGTATGCGGCGACCGAGTTG
CTCTTGCCCGGCGTCAATACGGGATAATACCGCGCCACATAGCAGAACTTTAAAAGTGCTCATCATTGGAAAACGTTCTTCG
GGGCGAAAACTCTCAAGGATCTTACCGCTGTTGAGATCCAGTTCGATGTAACCCACTCGTGCACCCAACTGATCTTCAGCAT
CTTTTACTTTCACCAGCGTTTCTGGGTGAGCAAAAACAGGAAGGCAAAATGCCGCAAAAAAGGGAATAAGGGCGACACGGAA
ATGTTGAATACTCATACTCTTCCTTTTTCAATATTATTGAAGCATTTATCAGGGTTATTGTCTCATGAGCGGATACATATTT
GAATGTATTTAGAAAAATAAACAAATAGGGGTTCCGCGCACATTTCCCCGAAAAGTGCCACCTGACGTCTAAGAAACCATTA
TTATCATGACATTAACCTATAAAAATAGGCGTATCACGAGGCCCTTTCGTC

>PTRB035 *Trichophyton rubrum* 'STRB012' anthranilate synthase component 2 with flanking regions; brown: pUC19, green: *Trp3*, blue: protospacer homologous

TCGCGCGTTTCGGTGATGACGGTGAAAACCTCTGACACATGCAGCTCCCGGAGACGGTCACAGCTTGTCTGTAAGCGGATGC
CGGGAGCAGACAAGCCCGTCAGGGCGCGTCAGCGGGTGTTGGCGGGTGTCGGGGCTGGCTTAACTATGCGGCATCAGAGCAG
ATTGTACTGAGAGTGCACCATATGCGGTGTGAAATACCGCACAGATGCGTAAGGAGAAAATACCGCATCAGGCGCCATTCGC
CATTCAGGCTGCGCAACTGTTGGGAAGGGCGATCGGTGCGGGCCTCTTCGCTATTACGCCAGCTGGCGAAAGGGGGATGTGC
TGCAAGGCGATTAAGTTGGGTAACGCCAGGGTTTTCCCAGTCACGACGTTGTAAAACGACGGCCAGTGAATTCGAGCTCGGT
ACCGAGCCAAGACAAGAGTACATCAGACGAGGGGATTGCCTCCGAGGATCTGACCTGAAGGAATAGGAGGCTCAATATCTGG
ACAAGTTCTTCAAGAGAACTTGCCTGCCAAGAGCTTTCCATCAACACATCAAGCCCAACCGAATTTGACCACCAACTACCAG
CTTCTAGACCTTTAACGCGTAGCCGAAAGGCCTCAATGCAGCTAGGAATGCTAATCTCGGTCTTTTCAACTTTCAATGTCCC
GCACTGGCCGTTGAGGATGGCGCGCCCCAGCTGGCTGGCCCCCACGATAAAGCTATCGCCAGACCCAGATAATTGTTCAAGT
TGTTGATCTTGCCGTGTATCCCGTAAGGATGTAGCTACCAAGAAATGGGCCAGTTTGAGTATGTACCTCTGCTCTGACAACC
AGGTATGAAACAAGATTTTCTGCCTTGTTTCTTTGGATTTTAACGTTGCTGGTGATTTAGACTGCGAACCGGCGTATTTGAG
AATGCTGGAAGCCTCGATTCCGTAGGACCCATGCGGCGACCTCCCAATCCCAACGGCATCATGCAAACTTGTGAGCTCCTCT
TCTGAAAAATGCGATAGAAGCTCATTTTCGCGTCGACGCTGCCAGGCTAACACGACTATGCGCAGCGCGGAAACCTCGTCGA
TGCTGGCCTCCTTGCTCAGCCATAGCGCATCGGACTTTATTTCGTCTGAATCTGAGTTGTTTTTAGAAGACGTGAAATGGAT
AGCGGCCGTCTTCGACTCAAATGCTGTCTTTGAAGCGCTGGAGGGCTTAGGGAAGGGGGAGTAAGGGCTTGAGAGGATGCGG
AGACCGGTTGAGGAAGAGAGGAAGTCGGAGATGTTCTTGTCAGGTTTAGACTGGTCGCCTGCCCTTGCTACAGACAAGTATG
CGGTTTTCCAGGATCTACAACTGCGGTTAGCATGCTGTTCAATTCCTGGGGTAAATTTAAAAAACGGGGGTATGCGAACATG
ATCTGGCTGTCGCCAAAGCACTCGTCCAGCGATGGGAAGTAGACGTCCGACGGCGGAGCCATGAGCGCATCCAGCTATATCA
TGGTCCGAGAGAGAGACCGAGGAGAGATCTGAGCCTGAGCAAGCACGCGGGCTGGTGCTAATTGATCATTCAATCTATAGGC
GGTGCTGGCTTGCTTTTGCGTGAAGTCGAAAAGTTGAAGCTCAAAAGGAAAAAAATAAGTCAACAAAGGTCGATGCCTATCT
GGGACTAGCGCCGATTTCAAAGCCGCCCTGCCTTATCTTTGAGTCTGGTCTTCTCGCATTCCCGCTTCGCCGTCTATATAAA
GAGAGGGACGGCGGACGGCGGGAGGACGACAGAATTCCCCTTCGACAGCAGAGACAGAGCAGAGACAGACGCAGCAGAAGCC
CGATAAACAGCCAGCAGAAGAGCAGAAAGAGAGCTATCACGATGGCGCTGGTTGACCATTCACCAAACCACCCCACCCCGTC
TGGACGGCTGGAGAACGCCTCCAATGTGATCCTGATCGATAACTACGACTCGTTTACATGGAATCTGTACCAGTATCTCGTT
CTCGAGGGTGCCACAGTCAGGGTTATCCGCAATGATGCCGCTACGCTGGAGGAGCTGATTGCTGAAAAGCCTACCCAGCTCG
TTCTCAGCCCCGGCCCTGGGCACCCCAAGACCGACGCAGGCATCTGCAACGAGGCCATCCAGCATTTTGCCGGCAAGATACC
TATTTTTGGAGTGTGCATGGGCCAGCAATGCATTATATCCTCGTTTGGGGGTGAGGTGGATGTCGCAGGAGAGATTCTGCAC
GGCAAGACATCTCCATTGAAGCATGACTCAAAGGGCGTATACGCCTCTCTTCCGGCTTCGCTGAACATTACACGCTACCATT
CGCTAGCTGGATCGGCCACCACCATCCCGGACTGTCTTGAGATATCCTCAACTACTGATTTGGGTGATCCTAACCGGCCTGA
CGTCATTATGGGCGTGAGACACAAGAAGTTCACCGTCGAGGGCGTTCAATTCCACCCAGAGAGCATTCTGACCGAACATGGG
CGAGCTATGTTCCGAAACTTCCTTCTGACTCGAGGGGGAACCTGGGAAGAGCACAATGCTTCTGCCCCTGGCCCAGCTACCG
TTCCATCTACAAACGGCCAATCTAGTGAAATGAAGAAGGGATCTATCCTCGATAAGATCTACGCTCACCGTCAAGCTGCAGT
TAAAGTGCAAAAGGAAATCCCATCTCAGCGTCCAGATGATCTTCAGGCTGCATACGACCTTGGTATTTCTCCTCCACAGATT
TCCTTCCCAGATCGCCTCGCCAAGTCTCCCTTCCCACTCTCTCTCATGGCCGAGATTAAGAGGGCTTCCCCTTCCAAGGGCA
TCATTGCCGCATCTATCTGTGCACCAGCTCAAGCCCGGAAGTACGCCATGGCCGGTGCCAGCGTTATCTCCGTGCTTACAGA
ACCAGAGTGGTTCAAGGGCAGCCTGGATGACCTACGTGCGGTCCGACAGAGTTTGGAGGGTATTCCTAATCGTCCTGCCATT
CTACGAAAGGAGTTTGTCTTTGATGAATACCAGATCCTTGAAGCCCGCCTTGCTGGTGCGGACACTGTGCTTTTGATCGTTA
AGATGCTTGCGGAGCCTCTCCTCAAGAGGCTGTTCGATTACTCTCGAAAGCTTGGTATGGAGCCTCTGGTCGAAGTCAACAA
CCCTGAGGAGATGGCAATTGCAGTTAGGCTGGGCTCCAAAGTCATTGGCGTCAACAACCGCAACCTCCAGAGTTTTGAGGTT
GATCTAGAGACAACCAGCCGTCTCATGGGACAAGTTCCGGAATCTACCATTGTCTGTGCCCTCAGTGGCATCTCAGGCCCCC
AGGATGTTGCACCATACCAGAAAAATGGAGTCAAGGCTGTTCTTGTTGGAGAGGCCCTTATGCGTGCTCAAGACGTTGGCGT
GTTTGTATCTAAGCTTTTTGGAACCAAACCTGGCCCCTTTGCCCAGACTCCTGGCGCTCCCTTGGTCAAAATTTGCGGAACG
AGATCTGCTGCTGCTGTCAAGGCAGCCATTGAAGGTGGTGCTGACCTGATTGGTATTATTCTTGCAGAAGGCCGATCAAGAA
CTGTGTCCACCGAGACTGCTCTGGAAATTTCGAAGACTGTCAAATCCACACCACGTCCCTCTTCCTTGAAGACCCAACCTCC
TGCTTATGGAGACGCATTCCTTGCCTCCAACTATTTTGACCACACTACTGGTCTCCTCAGAAACCCTGACCGGGCTCTTCTG
GTTGGAGTATTCCAGAACCAGCCTCTTTCCTATATAGTGGCTCAGCAGCAGAAACTAGACCTTGACGTTATCCAGCTCCACG
GATCGGAACCCGTTGAATGGCCATCGCTCCTCCCTGTCCCAGTTATCAAGAAGTTCTCTCCATCAGATTTGGGAATTTCTCG
CCGAGGCTATCACAGCCTTCCCCTCTTGGACTCTGGGGCTGGTGGTACTGGTGAGAGACTTGCCCTAGAACAAGTCAGGGGT
GTTCTAAAGAAAGACCCTGGACAACGAATTATCCTGGCTGGTGGCCTCGATGATAAGAATGTGACCGATGTTCTTCGCGCCC
TTGGCGAGGAAGGAAACAAGGTTGTGGGTGTGGACGTCAGCAGCGGCGTTGAGACTGACGGAGCACAGGATATTAAAAAGAT
CAAGGCTTTTATCACTGCTGCCAAAAACATTAGAAATACCACCTTGTGAATTCAGGGTGCTGACTACTTTCTAAATCAGTGA
TTATCATTTCGGGTGATAAAGGCCGTTGTTTTGGTTGAACTGCTTCGTTTACGTATATGCTTCTATGATAATGAGATGAGGT
TAATGTCATTAGAACATAGGGGGATATATATGATTCGAGCTTATTTCCCACCACTTAATCATGCAAGTGATGCCTCTGTTGA
TCTAACGACTACTTAATCCTCGGTTATCCCTATTTCTGTCACAATCTGCTTCTTTGGCTTCATATATATATCTTCAACGACA
CACTTAAGGATCCAACTAAGAAGTTCTCGCAGATTCCCTCAACGGAAATACGCCTGCAGGCATGCAAGCTTGGCGTAATCAT
GGTCATAGCTGTTTCCTGTGTGAAATTGTTATCCGCTCACAATTCCACACAACATACGAGCCGGAAGCATAAAGTGTAAAGC
CTGGGGTGCCTAATGAGTGAGCTAACTCACATTAATTGCGTTGCGCTCACTGCCCGCTTTCCAGTCGGGAAACCTGTCGTGC
CAGCTGCATTAATGAATCGGCCAACGCGCGGGGAGAGGCGGTTTGCGTATTGGGCGCTCTTCCGCTTCCTCGCTCACTGACT

CGCTGCGCTCGGTCGTTCGGCTGCGGCGAGCGGTATCAGCTCACTCAAAGGCGGTAATACGGTTATCCACAGAATCAGGGGA
TAACGCAGGAAAGAACATGTGAGCAAAAGGCCAGCAAAAGGCCAGGAACCGTAAAAAGGCCGCGTTGCTGGCGTTTTTCCAT
AGGCTCCGCCCCCCTGACGAGCATCACAAAAATCGACGCTCAAGTCAGAGGTGGCGAAACCCGACAGGACTATAAAGATACC
AGGCGTTTCCCCCTGGAAGCTCCCTCGTGCGCTCTCCTGTTCCGACCCTGCCGCTTACCGGATACCTGTCCGCCTTTCTCCC
TTCGGGAAGCGTGGCGCTTTCTCATAGCTCACGCTGTAGGTATCTCAGTTCGGTGTAGGTCGTTCGCTCCAAGCTGGGCTGT
GTGCACGAACCCCCCGTTCAGCCCGACCGCTGCGCCTTATCCGGTAACTATCGTCTTGAGTCCAACCCGGTAAGACACGACT
TATCGCCACTGGCAGCAGCCACTGGTAACAGGATTAGCAGAGCGAGGTATGTAGGCGGTGCTACAGAGTTCTTGAAGTGGTG
GCCTAACTACGGCTACACTAGAAGAACAGTATTTGGTATCTGCGCTCTGCTGAAGCCAGTTACCTTCGGAAAAAGAGTTGGT
AGCTCTTGATCCGGCAAACAAACCACCGCTGGTAGCGGTGGTTTTTTTGTTTGCAAGCAGCAGATTACGCGCAGAAAAAAAG
GATCTCAAGAAGATCCTTTGATCTTTTCTACGGGGTCTGACGCTCAGTGGAACGAAAACTCACGTTAAGGGATTTTGGTCAT
GAGATTATCAAAAAGGATCTTCACCTAGATCCTTTTAAATTAAAAATGAAGTTTTAAATCAATCTAAAGTATATATGAGTAA
ACTTGGTCTGACAGTTACCAATGCTTAATCAGTGAGGCACCTATCTCAGCGATCTGTCTATTTCGTTCATCCATAGTTGCCT
GACTCCCCGTCGTGTAGATAACTACGATACGGGAGGGCTTACCATCTGGCCCCAGTGCTGCAATGATACCGCGAGACCCACG
CTCACCGGCTCCAGATTTATCAGCAATAAACCAGCCAGCCGGAAGGGCCGAGCGCAGAAGTGGTCCTGCAACTTTATCCGCC
TCCATCCAGTCTATTAATTGTTGCCGGGAAGCTAGAGTAAGTAGTTCGCCAGTTAATAGTTTGCGCAACGTTGTTGCCATTG
CTACAGGCATCGTGGTGTCACGCTCGTCGTTTGGTATGGCTTCATTCAGCTCCGGTTCCCAACGATCAAGGCGAGTTACATG
ATCCCCCATGTTGTGCAAAAAAGCGGTTAGCTCCTTCGGTCCTCCGATCGTTGTCAGAAGTAAGTTGGCCGCAGTGTTATCA
CTCATGGTTATGGCAGCACTGCATAATTCTCTTACTGTCATGCCATCCGTAAGATGCTTTTCTGTGACTGGTGAGTACTCAA
CCAAGTCATTCTGAGAATAGTGTATGCGGCGACCGAGTTGCTCTTGCCCGGCGTCAATACGGGATAATACCGCGCCACATAG
CAGAACTTTAAAAGTGCTCATCATTGGAAAACGTTCTTCGGGGCGAAAACTCTCAAGGATCTTACCGCTGTTGAGATCCAGT
TCGATGTAACCCACTCGTGCACCCAACTGATCTTCAGCATCTTTTACTTTCACCAGCGTTTCTGGGTGAGCAAAAACAGGAA
GGCAAAATGCCGCAAAAAAGGGAATAAGGGCGACACGGAAATGTTGAATACTCATACTCTTCCTTTTTCAATATTATTGAAG
CATTTATCAGGGTTATTGTCTCATGAGCGGATACATATTTGAATGTATTTAGAAAAATAAACAAATAGGGGTTCCGCGCACA
TTTCCCCGAAAAGTGCCACCTGACGTCTAAGAAACCATTATTATCATGACATTAACCTATAAAAATAGGCGTATCACGAGGC
CCTTTCGTC

>PTRB042 *Trichophyton rubrum Trp3* sgRNA with T7 promoter construct 1; brown: pUC19, red: T7 promoter, blue: protospacer homologous, green: sgRNA tail, underline: primer homologous
TCGCGCGTTTCGGTGATGACGGTGAAAACCTCTGACACATGCAGCTCCCGGAGACGGTCACAGCTTGTCTGTAAGCGGATGC
CGGGAGCAGACAAGCCCGTCAGGGCGCGTCAGCGGGTGTTGGCGGGTGTCGGGGCTGGCTTAACTATGCGGCATCAGAGCAG
ATTGTACTGAGAGTGCACCATATGCGGTGTGAAATACCGCACAGATGCGTAAGGAGAAAATACCGCATCAGGCGCCATTCGC
CATTCAGGCTGCGCAACTGTTGGGAAGGGCGATCGGTGCGGGCCTCTTCGCTATTACGCCAGCTGGCGAAAGGGGGATGTGC
TGCAAGGCGATTAAGTTGGGTAACGCCAGGGTTTTCCCAGTCACGACGTTGTAAAACGACGGCCAGTGAATTCGAGCTCGGT
ACCCGGGGATCCTCTAGAGTCGACCTGCAGGTAATACGACTCACTATAGGAGAGCATTCTGACCGAACAGTTTTAGAGCTAG
AAATAGCAAGTTAAAATAAGGCTAGTCCGTTATCAACTTGAAAAAGTGGCACCGAGTCGGTGCTTTTGGCCGGCATGGTCCC
AGCCTCCTCGCTGGCGCCGGCTGGGCAACATGCTTCGGCATGGCGAATGGGACTGATTTAATAGCTCCATGTCAACAAGAAT
AAAACGCGTTTCGGGTTTACCTCTTCCAGATACAGCTCATCTGCAATGCATTAATGCATTGGACCTCGCAACCCTAGTACGC
CCTTCAGGCTCCGGCGAAGCAGAAGAATAGCTTAGCAGAGTCTATTTTCATTTTCGGGAGACGAGATCAAGCAGATCAACGG
TCGTCAAGAGACCTACGAGACTGAGGAATCCGCTCTTGGCTCGCGGCCGCCGAGACAGCAGAATCACCGCCCAAGTTAAGCC
TTTGTGCTGATCATGCTCTCGAACGAAGCTTGGCGTAATCATGGTCATAGCTGTTTCCTGTGTGAAATTGTTATCCGCTCAC
AATTCCACACAACATACGAGCCGGAAGCATAAAGTGTAAAGCCTGGGGTGCCTAATGAGTGAGCTAACTCACATTAATTGCG
TTGCGCTCACTGCCCGCTTTCCAGTCGGGAAACCTGTCGTGCCAGCTGCATTAATGAATCGGCCAACGCGCGGGGAGAGGCG
GTTTGCGTATTGGGCGCTCTTCCGCTTCCTCGCTCACTGACTCGCTGCGCTCGGTCGTTCGGCTGCGGCGAGCGGTATCAGC
TCACTCAAAGGCGGTAATACGGTTATCCACAGAATCAGGGGATAACGCAGGAAAGAACATGTGAGCAAAAGGCCAGCAAAAG
GCCAGGAACCGTAAAAAGGCCGCGTTGCTGGCGTTTTTCCATAGGCTCCGCCCCCCTGACGAGCATCACAAAAATCGACGCT
CAAGTCAGAGGTGGCGAAACCCGACAGGACTATAAAGATACCAGGCGTTTCCCCCTGGAAGCTCCCTCGTGCGCTCTCCTGT
TCCGACCCTGCCGCTTACCGGATACCTGTCCGCCTTTCTCCCTTCGGGAAGCGTGGCGCTTTCTCATAGCTCACGCTGTAGG
TATCTCAGTTCGGTGTAGGTCGTTCGCTCCAAGCTGGGCTGTGTGCACGAACCCCCCGTTCAGCCCGACCGCTGCGCCTTAT
CCGGTAACTATCGTCTTGAGTCCAACCCGGTAAGACACGACTTATCGCCACTGGCAGCAGCCACTGGTAACAGGATTAGCAG
AGCGAGGTATGTAGGCGGTGCTACAGAGTTCTTGAAGTGGTGGCCTAACTACGGCTACACTAGAAGAACAGTATTTGGTATC
TGCGCTCTGCTGAAGCCAGTTACCTTCGGAAAAAGAGTTGGTAGCTCTTGATCCGGCAAACAAACCACCGCTGGTAGCGGTG
GTTTTTTTGTTTGCAAGCAGCAGATTACGCGCAGAAAAAAAGGATCTCAAGAAGATCCTTTGATCTTTTCTACGGGGTCTGA
CGCTCAGTGGAACGAAAACTCACGTTAAGGGATTTTGGTCATGAGATTATCAAAAAGGATCTTCACCTAGATCCTTTTAAAT
TAAAAATGAAGTTTTAAATCAATCTAAAGTATATATGAGTAAACTTGGTCTGACAGTTACCAATGCTTAATCAGTGAGGCAC
CTATCTCAGCGATCTGTCTATTTCGTTCATCCATAGTTGCCTGACTCCCCGTCGTGTAGATAACTACGATACGGGAGGGCTT
ACCATCTGGCCCCAGTGCTGCAATGATACCGCGAGACCCACGCTCACCGGCTCCAGATTTATCAGCAATAAACCAGCCAGCC
GGAAGGGCCGAGCGCAGAAGTGGTCCTGCAACTTTATCCGCCTCCATCCAGTCTATTAATTGTTGCCGGGAAGCTAGAGTAA

GTAGTTCGCCAGTTAATAGTTTGCGCAACGTTGTTGCCATTGCTACAGGCATCGTGGTGTCACGCTCGTCGTTTGGTATGGC
TTCATTCAGCTCCGGTTCCCAACGATCAAGGCGAGTTACATGATCCCCCATGTTGTGCAAAAAAGCGGTTAGCTCCTTCGGT
CCTCCGATCGTTGTCAGAAGTAAGTTGGCCGCAGTGTTATCACTCATGGTTATGGCAGCACTGCATAATTCTCTTACTGTCA
TGCCATCCGTAAGATGCTTTTCTGTGACTGGTGAGTACTCAACCAAGTCATTCTGAGAATAGTGTATGCGGCGACCGAGTTG
CTCTTGCCCGGCGTCAATACGGGATAATACCGCGCCACATAGCAGAACTTTAAAAGTGCTCATCATTGGAAAACGTTCTTCG
GGGCGAAAACTCTCAAGGATCTTACCGCTGTTGAGATCCAGTTCGATGTAACCCACTCGTGCACCCAACTGATCTTCAGCAT
CTTTTACTTTCACCAGCGTTTCTGGGTGAGCAAAAACAGGAAGGCAAAATGCCGCAAAAAAGGGAATAAGGGCGACACGGAA
ATGTTGAATACTCATACTCTTCCTTTTTCAATATTATTGAAGCATTTATCAGGGTTATTGTCTCATGAGCGGATACATATTT
GAATGTATTTAGAAAAATAAACAAATAGGGGTTCCGCGCACATTTCCCCGAAAAGTGCCACCTGACGTCTAAGAAACCATTA
TTATCATGACATTAACCTATAAAAATAGGCGTATCACGAGGCCCTTTCGTC

>PTRB043 *Trichophyton rubrum Trp3* sgRNA with T7 promoter construct 2; brown: pUC19, red: T7 promoter, blue: protospacer homologous, green: sgRNA tail, underline: primer homologous

TCGCGCGTTTCGGTGATGACGGTGAAAACCTCTGACACATGCAGCTCCCGGAGACGGTCACAGCTTGTCTGTAAGCGGATGC
CGGGAGCAGACAAGCCCGTCAGGGCGCGTCAGCGGGTGTTGGCGGGTGTCGGGGCTGGCTTAACTATGCGGCATCAGAGCAG
ATTGTACTGAGAGTGCACCATATGCGGTGTGAAATACCGCACAGATGCGTAAGGAGAAAATACCGCATCAGGCGCCATTCGC
CATTCAGGCTGCGCAACTGTTGGGAAGGGCGATCGGTGCGGGCCTCTTCGCTATTACGCCAGCTGGCGAAAGGGGGATGTGC
TGCAAGGCGATTAAGTTGGGTAACGCCAGGGTTTTCCCAGTCACGACGTTGTAAAACGACGGCCAGTGAATTCGAGCTCGGT
ACCCGGGGATCCTCTAGAGTCGACCTGCAGGTAATACGACTCACTATAGGCCAATCTAGTGAAATGAAGAGTTTTAGAGCTA
GAAATAGCAAGTTAAAATAAGGCTAGTCCGTTATCAACTTGAAAAAGTGGCACCGAGTCGGTGCTTTTGGCCGGCATGGTCC
CAGCCTCCTCGCTGGCGCCGGCTGGGCAACATGCTTCGGCATGGCGAATGGGACTGATTTAATAGCTCCATGTCAACAAGAA
TAAAACGCGTTTCGGGTTTACCTCTTCCAGATACAGCTCATCTGCAATGCATTAATGCATTGGACCTCGCAACCCTAGTACG
CCCTTCAGGCTCCGGCGAAGCAGAAGAATAGCTTAGCAGAGTCTATTTTCATTTTCGGGAGACGAGATCAAGCAGATCAACG
GTCGTCAAGAGACCTACGAGACTGAGGAATCCGCTCTTGGCTCGCGGCCGCCGAGACAGCAGAATCACCGCCCAAGTTAAGC
CTTTGTGCTGATCATGCTCTCGAACGAAGCTTGGCGTAATCATGGTCATAGCTGTTTCCTGTGTGAAATTGTTATCCGCTCA
CAATTCCACACAACATACGAGCCGGAAGCATAAAGTGTAAAGCCTGGGGTGCCTAATGAGTGAGCTAACTCACATTAATTGC
GTTGCGCTCACTGCCCGCTTTCCAGTCGGGAAACCTGTCGTGCCAGCTGCATTAATGAATCGGCCAACGCGCGGGGAGAGGC
GGTTTGCGTATTGGGCGCTCTTCCGCTTCCTCGCTCACTGACTCGCTGCGCTCGGTCGTTCGGCTGCGGCGAGCGGTATCAG
CTCACTCAAAGGCGGTAATACGGTTATCCACAGAATCAGGGGATAACGCAGGAAAGAACATGTGAGCAAAAGGCCAGCAAAA
GGCCAGGAACCGTAAAAAGGCCGCGTTGCTGGCGTTTTTCCATAGGCTCCGCCCCCCTGACGAGCATCACAAAAATCGACGC
TCAAGTCAGAGGTGGCGAAACCCGACAGGACTATAAAGATACCAGGCGTTTCCCCCTGGAAGCTCCCTCGTGCGCTCTCCTG
TTCCGACCCTGCCGCTTACCGGATACCTGTCCGCCTTTCTCCCTTCGGGAAGCGTGGCGCTTTCTCATAGCTCACGCTGTAG
GTATCTCAGTTCGGTGTAGGTCGTTCGCTCCAAGCTGGGCTGTGTGCACGAACCCCCCGTTCAGCCCGACCGCTGCGCCTTA
TCCGGTAACTATCGTCTTGAGTCCAACCCGGTAAGACACGACTTATCGCCACTGGCAGCAGCCACTGGTAACAGGATTAGCA
GAGCGAGGTATGTAGGCGGTGCTACAGAGTTCTTGAAGTGGTGGCCTAACTACGGCTACACTAGAAGAACAGTATTTGGTAT
CTGCGCTCTGCTGAAGCCAGTTACCTTCGGAAAAAGAGTTGGTAGCTCTTGATCCGGCAAACAAACCACCGCTGGTAGCGGT
GGTTTTTTTGTTTGCAAGCAGCAGATTACGCGCAGAAAAAAAGGATCTCAAGAAGATCCTTTGATCTTTTCTACGGGGTCTG
ACGCTCAGTGGAACGAAAACTCACGTTAAGGGATTTTGGTCATGAGATTATCAAAAAGGATCTTCACCTAGATCCTTTTAAA
TTAAAAATGAAGTTTTAAATCAATCTAAAGTATATATGAGTAAACTTGGTCTGACAGTTACCAATGCTTAATCAGTGAGGCA
CCTATCTCAGCGATCTGTCTATTTCGTTCATCCATAGTTGCCTGACTCCCCGTCGTGTAGATAACTACGATACGGGAGGGCT
TACCATCTGGCCCCAGTGCTGCAATGATACCGCGAGACCCACGCTCACCGGCTCCAGATTTATCAGCAATAAACCAGCCAGC
CGGAAGGGCCGAGCGCAGAAGTGGTCCTGCAACTTTATCCGCCTCCATCCAGTCTATTAATTGTTGCCGGGAAGCTAGAGTA
AGTAGTTCGCCAGTTAATAGTTTGCGCAACGTTGTTGCCATTGCTACAGGCATCGTGGTGTCACGCTCGTCGTTTGGTATGG
CTTCATTCAGCTCCGGTTCCCAACGATCAAGGCGAGTTACATGATCCCCCATGTTGTGCAAAAAAGCGGTTAGCTCCTTCGG
TCCTCCGATCGTTGTCAGAAGTAAGTTGGCCGCAGTGTTATCACTCATGGTTATGGCAGCACTGCATAATTCTCTTACTGTC
ATGCCATCCGTAAGATGCTTTTCTGTGACTGGTGAGTACTCAACCAAGTCATTCTGAGAATAGTGTATGCGGCGACCGAGTT
GCTCTTGCCCGGCGTCAATACGGGATAATACCGCGCCACATAGCAGAACTTTAAAAGTGCTCATCATTGGAAAACGTTCTTC
GGGGCGAAAACTCTCAAGGATCTTACCGCTGTTGAGATCCAGTTCGATGTAACCCACTCGTGCACCCAACTGATCTTCAGCA
TCTTTTACTTTCACCAGCGTTTCTGGGTGAGCAAAAACAGGAAGGCAAAATGCCGCAAAAAAGGGAATAAGGGCGACACGGA
AATGTTGAATACTCATACTCTTCCTTTTTCAATATTATTGAAGCATTTATCAGGGTTATTGTCTCATGAGCGGATACATATT
TGAATGTATTTAGAAAAATAAACAAATAGGGGTTCCGCGCACATTTCCCCGAAAAGTGCCACCTGACGTCTAAGAAACCATT
ATTATCATGACATTAACCTATAAAAATAGGCGTATCACGAGGCCCTTTCGTC

>NTRB060 *Ura3* with flanking region; EcoRI
CAGGTTCACATTTCGAGAATTCCAG

>NTRB061 *Ura3* with flanking region; BamHI
CATATGGATCCAGGGAGAGCACAGCTAACTTGAC

>NTRB076 *Trp3* with SdaI restriction site
GAGACCTGCAGGAGCCAAGACAAGAGTACATCAGACG

>NTRB077 *Trp3* with KpnI restriction site
CTCTGGTACCGGCGTATTTCCGTTGAGGGAATCTGC

>NTRB80 DNA fragment for T7 RNA transcript
GCAGGTAATACGACTCACTATAG

>NTRB81 DNA fragment for T7 RNA transcript
AAAAGCACCGACTCGGTG

>NTRB85 orotidine 5-phosphate decarboxylase with flanking regions
GTCCCTTCCACTGTTCCTCGAC

>NTRB86 orotidine 5-phosphate decarboxylase with flanking regions
GAGCACAGCTAACTTGACATCTGC

>NTRB115 *Trp3* sequencing primer
GACGCAGGCATCTGCAACGAG

>NTRB116 *Trp3* sequencing primer
CACAGTGTCCGCACCAGCAAG

>NTRB107 sgRNA with T7 promoter, TrpC construct 1
TGTTCGGTCAGAATGCTCTCCTATAGTGAGTCGTATTACCTGCAG

>NTRB108 sgRNA with T7 promoter, TrpC construct 1
GGAGAGCATTCTGACCGAACAGTTTTAGAGCTAGAAATAGCAAGTTAAAATAAGGC

>NTRB109 sgRNA with T7 promoter, TrpC construct 2
TCTTCATTTCACTAGATTGGCCTATAGTGAGTCGTATTACCTGCAG

>NTRB110 sgRNA with T7 promoter, TrpC construct 2
GGCCAATCTAGTGAAATGAAGAGTTTTAGAGCTAGAAATAGCAAGTTAAAATAAGGC

>NR_131330.1 *Trichophyton rubrum* CBS 392.58 ITS region; from TYPE material
AAGTAAAAGTCGTAACAAGGTTTCCGTAGGTGAACCTGCGGAAGGATCATTAACGCGCAGGCCGGAGGCTGGCCCCCCACGA
TAGGGACCGACGTTCCATCAGGGGTGAGCAGACGTGCGCCGGCCGTACGCCCCCATTCTTGTCTACCTCACCCGGTTGCCTC
GGCGGGCCGCGCTCCCCCTGCCAGGGAGAGCCGTCCGGCGGGCCCCTTCTGGGAGCCTCGAGCCGGACCGCGCCCGCCGGAG
GACAGACACCAAGAAAAAATTCTCTGAAGAGCTGTCAGTCTGAGCGTTTAGCAAGCACAATCAGTTAAAACTTTCAACAACG
GATCTCTTGGTTCCGGCATCGATGAAGAACGCAGCGAAATGCGATAAGTAATGTGAATTGCAGAATTCCGTGAATCATCGAA
TCTTTGAACGCACATTGCGCCCTCTGGCATTCCGGGGGGCATGCCTGTTCGAGCGTCATTTCAACCCCTCAAGCCCGGCTTG
TGTGATGGACGACCGTCCGGCCCCTCCCTTCGGGGGCGGGACGCGCCCGAAAAGCAGTGGCCAGGCCGCGATTCCGGCTTCC
TAGGCGAATGGGCAGCCAATTCAGCGCCCTCAGGACCGGCCGCCCTGGCCCCAATCTTTATATATATATATATCTTTTCAGG
TTGACCTCGGATCAGGTAGGGATACCCGCTGAACTTAAGCATATCAATAAGCGGAGGAAAAGAAACCAACC

>*Trichophyton rubrum* STRB012
AAGTAAAAGTCGTAACAAGGTTTCCGTAGGTGAACCTGCGGAAGGATCATTAACGCGCAGGCCGGAGGCTGGCCCCCCACGA
TAGGGACCGACGTTCCATCAGGGGTGAGCAGACGTGCGCCGGCCGTACGCCCCCATTCTTGTCTACCTCACCCGGTTGCCTC
GGCGGGCCGCGCTCCCCCTGCCAGGGAGAGCCGTCCGGCGGGCCCCTTTTGGGAGCCTCGAGCCGGACCGCGCCCGCCGGAG
GACAGACACCAAGAAAAAATTCTCTGAAGAGCTGTCAGTCTGAGCGTTTAGCAAGCACAATCAGTTAAAACTTTCAACAACG

GATCTCTTGGTTCCGGCATCGATGAAGAACGCAGCGAAATGCGATAAGTAATGTGAATTGCAGAATTCCGTGAATCATCGAA
TCTTTGAACGCACATTGCGCCCTCTGGCATTCCGGGGGGGCATGCCTGTTCGAGCGTCATTTCAACCCCTCAAGCCCGGCTT
GTGTGATGGACGACCGTCCGGCCCCTCCCTTCGGGGGCGGGACGCGCCCGAAAAGCAGTGGCCAGGCCGCGATTCCGGCTTC
CTAGGCGAATGGGCAGCCAATTCAGCGCCCTCAGGACCGGCCGCCCTGGCCCCAATCTTTATATATATATATATCTTTTCAG
GTTGACCTCGGATCAGGTAGGGATACCCGCTGAACTTAAGCATATCAATAAGCGGAGGAAAAGAAACCAACA

>*Trichophyton rubrum* STRB008
AAGTAAAAGTCGTAACAAGGTTTCCGTAGGTGAACCTGCGGAAGGATCATTAACGCGCAGGCCGGAGGCTGGCCCCCCACGA
TAGGGACCGACGTTCCATCAGGGGTGAGCAGACGTGCGCCGGCCGTACGCCCCCATTCTTGTCTACCTCACCCGGTTGCCTC
GGCGGGCCGCGCTCCCCCTGCCAGGGAGAGCCGTCCGGCGGGCCCCTTCTGGGAGCCTCGAGCCGGACCGCGCCCGCCGGAG
GACAGACACCAAGAAAAAATTCTCTGAAGAGCTGTCAGTCTGAGCGTTTAGCAAGCACAATCAGTTAAAACTTTCAACAACG
GATCTCTTGGTTCCGGCATCGATGAAGAACGCAGCGAAATGCGATAAGTAATGTGAATTGCAGAATTCCGTGAATCATCGAA
TCTTTGAACGCACATTGCGCCCTCTGGCATTCCGGGGGGCATGCCTGTTCGAGCGTCATTTCAACCCCTCAAGCCCGGCTTG
TGTGATGGACGACCGTCCGGCCCCTCCCTTCGGGGGCGGGACGCGCCCGAAAAGCAGTGGCCAGGCCGCGATTCCGGCTTCC
TAGGCGAATGGGCAGCCAATTCAGCGCCCTCAGGACCGGCCGCCCTGGCCCCAATCTTTATATATATATATATCTTTTCAGG
TTGACCTCGGATCAGGTAGGGATACCCGCTGAACTTAAGCATATCAATAAGCGGAGGAAAAGAAACCAACA

CLUSTAL 2.1 multiple sequence alignment

```
STRB012         AAGTAAAAGTCGTAACAAGGTTTCCGTAGGTGAACCTGCGGAAGGATCATTAACGCGCAG
STRB008         AAGTAAAAGTCGTAACAAGGTTTCCGTAGGTGAACCTGCGGAAGGATCATTAACGCGCAG
CBS_392_58      AAGTAAAAGTCGTAACAAGGTTTCCGTAGGTGAACCTGCGGAAGGATCATTAACGCGCAG
                ************************************************************

STRB012         GCCGGAGGCTGGCCCCCCACGATAGGGACCGACGTTCCATCAGGGGTGAGCAGACGTGCG
STRB008         GCCGGAGGCTGGCCCCCCACGATAGGGACCGACGTTCCATCAGGGGTGAGCAGACGTGCG
CBS_392_58      GCCGGAGGCTGGCCCCCCACGATAGGGACCGACGTTCCATCAGGGGTGAGCAGACGTGCG
                ************************************************************

STRB012         CCGGCCGTACGCCCCCATTCTTGTCTACCTCACCCGGTTGCCTCGGCGGGCCGCGCTCCC
STRB008         CCGGCCGTACGCCCCCATTCTTGTCTACCTCACCCGGTTGCCTCGGCGGGCCGCGCTCCC
CBS_392_58      CCGGCCGTACGCCCCCATTCTTGTCTACCTCACCCGGTTGCCTCGGCGGGCCGCGCTCCC
                ************************************************************

STRB012         CCTGCCAGGGAGAGCCGTCCGGCGGGCCCCTTTTGGGAGCCTCGAGCCGGACCGCGCCCG
STRB008         CCTGCCAGGGAGAGCCGTCCGGCGGGCCCCTTCTGGGAGCCTCGAGCCGGACCGCGCCCG
CBS_392_58      CCTGCCAGGGAGAGCCGTCCGGCGGGCCCCTTCTGGGAGCCTCGAGCCGGACCGCGCCCG
                ******************************** ***************************

STRB012         CCGGAGGACAGACACCAAGAAAAAATTCTCTGAAGAGCTGTCAGTCTGAGCGTTTAGCAA
STRB008         CCGGAGGACAGACACCAAGAAAAAATTCTCTGAAGAGCTGTCAGTCTGAGCGTTTAGCAA
CBS_392_58      CCGGAGGACAGACACCAAGAAAAAATTCTCTGAAGAGCTGTCAGTCTGAGCGTTTAGCAA
                ************************************************************

STRB012         GCACAATCAGTTAAAACTTTCAACAACGGATCTCTTGGTTCCGGCATCGATGAAGAACGC
STRB008         GCACAATCAGTTAAAACTTTCAACAACGGATCTCTTGGTTCCGGCATCGATGAAGAACGC
CBS_392_58      GCACAATCAGTTAAAACTTTCAACAACGGATCTCTTGGTTCCGGCATCGATGAAGAACGC
                ************************************************************

STRB012         AGCGAAATGCGATAAGTAATGTGAATTGCAGAATTCCGTGAATCATCGAATCTTTGAACG
STRB008         AGCGAAATGCGATAAGTAATGTGAATTGCAGAATTCCGTGAATCATCGAATCTTTGAACG
CBS_392_58      AGCGAAATGCGATAAGTAATGTGAATTGCAGAATTCCGTGAATCATCGAATCTTTGAACG
                ************************************************************

STRB012         CACATTGCGCCCTCTGGCATTCCGGGGGGGCATGCCTGTTCGAGCGTCATTTCAACCCCT
STRB008         CACATTGCGCCCTCTGGCATTCCGGGGGG-CATGCCTGTTCGAGCGTCATTTCAACCCCT
CBS_392_58      CACATTGCGCCCTCTGGCATTCCGGGGGG-CATGCCTGTTCGAGCGTCATTTCAACCCCT
```

```
                ***************************** ******************************

STRB012         CAAGCCCGGCTTGTGTGATGGACGACCGTCCGGCCCCTCCCTTCGGGGGCGGGACGCGCC
STRB008         CAAGCCCGGCTTGTGTGATGGACGACCGTCCGGCCCCTCCCTTCGGGGGCGGGACGCGCC
CBS_392_58      CAAGCCCGGCTTGTGTGATGGACGACCGTCCGGCCCCTCCCTTCGGGGGCGGGACGCGCC
                ************************************************************

STRB012         CGAAAAGCAGTGGCCAGGCCGCGATTCCGGCTTCCTAGGCGAATGGGCAGCCAATTCAGC
STRB008         CGAAAAGCAGTGGCCAGGCCGCGATTCCGGCTTCCTAGGCGAATGGGCAGCCAATTCAGC
CBS_392_58      CGAAAAGCAGTGGCCAGGCCGCGATTCCGGCTTCCTAGGCGAATGGGCAGCCAATTCAGC
                ************************************************************

STRB012         GCCCTCAGGACCGGCCGCCCTGGCCCCAATCTTTATATATATATATATATCTTTTCAGGTTG
STRB008         GCCCTCAGGACCGGCCGCCCTGGCCCCAATCTTTATATATATATATATATCTTTTCAGGTTG
CBS_392_58      GCCCTCAGGACCGGCCGCCCTGGCCCCAATCTTTATATATATATATATATCTTTTCAGGTTG
                ************************************************************

STRB012         ACCTCGGATCAGGTAGGGATACCCGCTGAACTTAAGCATATCAATAAGCGGAGGAAAAGA
STRB008         ACCTCGGATCAGGTAGGGATACCCGCTGAACTTAAGCATATCAATAAGCGGAGGAAAAGA
CBS_392_58      ACCTCGGATCAGGTAGGGATACCCGCTGAACTTAAGCATATCAATAAGCGGAGGAAAAGA
                ************************************************************

STRB012         AACCAACA
STRB008         AACCAACA
CBS_392_58      AACCAACC
                *******
```
