# Supplementary material for: Auxotrophic mutations of Trichophyton rubrum created by in vitro synthesized Cas9 ribonucleoprotein
Source: BMC Biotechnol. 2020 Jan 20;20:6. doi: 10.1186/s12896-020-0601-z (PMC6971929; doi:10.1186/s12896-020-0601-z)
Supplement: Supplementary file 2 — Additional file 2. Supplement_media; cultivation media. [file 12896_2020_601_MOESM2_ESM.pdf]

Trichophyton Agar Nr. 1 (Tr1)

Dextrose	40g
Casamino Acids	2.5g
Monopotassium Phosphate	1.8g
Magnesium Sulfate	0.1g
Agar	18g

pH 5.8

for Uracil auxotrophic strains add 0.16g Uracil and 0.08g Uridine
for Tryptophan auxotrophic strains add 0.16g Tryptophan

Potato dextrose agar (PDA)

Potato (boiled and filtrated)	200g
Dextrose	20g
Agar	18g

Synthetic Complete (SC/SD)

Bact-yeast nitrogen base*	6.7g
Glucose	20g
Agar	20g
drop-out mix	2g

pH 5.8

* without amino acids (Difco 291920)
for Uracil auxotrophic strains add additional 0.08g Uracil and 0.08g Uridine
for Tryptophan auxotrophic strains add additional 0.08g Tryptophan

Drop-out mix for 1 l

Adenine	0.02 g
Alanine	0.08 g
Arginine	0.08 g
Asparagine	0.08 g
Aspartic-acid	0.08 g
Cysteine	0.08 g
Glutamine	0.08 g
Glutamic-acid	0.08 g
Glycine	0.08 g
Histidine	0.08 g

Inositol	0.08 g
Isoleucine	0.08 g
Leucine	0.4 g
Lysine	0.08 g
Methionine	0.08 g
para-Aminobenzoic-acid	0.008 g
Phenylalanine	0.08 g
Proline	0.08 g
Serine	0.08 g
Threonine	0.08 g
Tryptophan	0.08 g
Tyrosine	0.08 g
Uracil	0.08 g
Valine	0.08 g
